# Geometry-complete perceptron networks for 3D molecular graphs

**DOI:** 10.1093/bioinformatics/btae087

**Published:** 2024-02-19

**Authors:** Alex Morehead, Jianlin Cheng

**Affiliations:** Electrical Engineering & Computer Science, University of Missouri-Columbia, Columbia, MO 65211, United States; Electrical Engineering & Computer Science, University of Missouri-Columbia, Columbia, MO 65211, United States

## Abstract

**Motivation:**

The field of geometric deep learning has recently had a profound impact on several scientific domains such as protein structure prediction and design, leading to methodological advancements within and outside of the realm of traditional machine learning. Within this spirit, in this work, we introduce GCPNet, a new chirality-aware SE(3)-equivariant graph neural network designed for representation learning of 3D biomolecular graphs. We show that GCPNet, unlike previous representation learning methods for 3D biomolecules, is widely applicable to a variety of invariant or equivariant node-level, edge-level, and graph-level tasks on biomolecular structures while being able to (1) learn important chiral properties of 3D molecules and (2) detect external force fields.

**Results:**

Across four distinct molecular-geometric tasks, we demonstrate that GCPNet’s predictions (1) for protein–ligand binding affinity achieve a statistically significant correlation of 0.608, more than 5%, greater than current state-of-the-art methods; (2) for protein structure ranking achieve statistically significant target-local and dataset-global correlations of 0.616 and 0.871, respectively; (3) for Newtownian many-body systems modeling achieve a task-averaged mean squared error less than 0.01, more than 15% better than current methods; and (4) for molecular chirality recognition achieve a state-of-the-art prediction accuracy of 98.7%, better than any other machine learning method to date.

**Availability and implementation:**

The source code, data, and instructions to train new models or reproduce our results are freely available at https://github.com/BioinfoMachineLearning/GCPNet.

## 1 Introduction

Over the last several years, the field of deep learning has pioneered many new methods designed to process graph-structured inputs. Being a ubiquitous form of information, graph-structured data arises from numerous sources such as the fields of physics and chemistry, e.g. in the form of interacting particle systems or molecular graphs. Moreover, the relational nature of graph-structured data allows one to identify and characterize topological associations between entities in large real-world networks (e.g. social networks).

In scientific domains such as computational biology and chemistry, graphs are often used to represent the 3D structures of molecules (Ma *et al.* 2022), chemical compounds (Wu *et al.* 2022), and even large biomolecules such as proteins (Karimi *et al.* 2019, Baldassarre *et al.* 2021, Xia and Ku 2021, Morehead *et al.* 2022b, Wang *et al.* 2023a). Underlying many of these successful examples of graph representations are graph neural networks (GNNs), a class of machine learning algorithms specialized in processing irregularly-structured input data such as graphs. Careful applications of GNNs in scientific domains have considered the physical symmetries present in many scientific data and have leveraged such symmetries to design new attention-based neural network architectures (Jumper *et al.* 2021, Morehead *et al.* 2022a).

Throughout their development, geometric deep learning methods have expanded to incorporate within them equivariance to various geometric symmetry groups to enhance their generalization capabilities and adversarial robustness. Methods such as group-equivariant CNNs (Cohen and Welling 2016), Tensor Field Networks (Thomas *et al.* 2018), and equivariant GNNs (Batzner *et al.* 2022) such as GVP-GNNs (Jing *et al.* 2020, 2021) and ClofNet (Du *et al.* 2022) have paved the way for the development of future deep learning models that respect physical symmetries present in 3D data (e.g. rotation equivariance with respect to input data symmetries).

Within this spirit, in this work, we introduce a new geometric GNN model, GCPNet, that is equivariant to the group of 3D rotations and translations (i.e. SE(3), the special Euclidean group, as studied in previous works (Fuchs *et al.* 2021)) and, uniquely, that simultaneously guarantees chirality sensitivity and geometric (vector) information completeness following graph message-passing on 3D point clouds. We demonstrate its expressiveness and flexibility for modeling physical systems through rigorous experiments for distinct molecular-geometric tasks. In detail, we provide the following contributions:

- In contrast to prior geometric networks for molecules that are insensitive to their chemical chirality (Jing *et al.* 2020, 2021), cannot detect global physical forces acting upon each atom (Wang *et al.* 2023b), or do not directly learn geometric features (Du *et al.* 2022), we present the first geometric GNN architecture with the following desirable properties for learning from 3D molecules as described in Supplementary Table S11: (1) the ability to directly predict translation and rotation-invariant scalar properties and rotation-equivariant vector-valued quantities for nodes and edges, respectively; (2) a rotation and translation-equivariant method for iteratively updating node positions in 3D space; (3) sensitivity to molecular chirality; and (4) a means by which to learn from and account for the global forces acting upon the atoms within its inputs.
- We establish new state-of-the-art results for four distinct molecular-geometric representation learning tasks—molecular chirality recognition, protein–ligand binding affinity (LBA) prediction, protein structure ranking (PSR), and Newtonian many-body-systems modeling—where model predictions vary from analyzing individual nodes to summarizing entire graph inputs. GCPNet’s performance for these tasks is statistically significant and surpasses that of previous state-of-the-art machine learning methods for 3D molecules.

## 2 Methods

### 2.1 Preliminaries

#### 2.1.1 Overview of the problem setting

We represent a 3D molecular structure (e.g. a protein or small molecule) as a 3D *k*-nearest neighbors (*k*-NN) graph G=(V,E) with V and E representing the graph’s set of nodes and set of edges, respectively, and N=|V| and E=|E| representing the number of nodes and the number of edges in the graph, respectively. In addition, $X\in R^{N\times 3}$ represents the respective Cartesian coordinates for each node. We then design E(3)-invariant (i.e. 3D rotation, reflection, and translation-invariant) node features $H\in R^{N\times h}$ and edge features $E\in R^{E\times e}$ as well as O(3)-equivariant (3D rotation and reflection-equivariant) node features $\chi \in R^{N\times (m\times 3)}$ and edge features $\xi \in R^{E\times (x\times 3)}$, respectively.

Upon constructing such features, we apply several layers of graph message-passing using a neural network Φ (which later on we refer to as GCPNet) that updates node and edge features using invariant and equivariant representations for the corresponding feature types. Importantly, Φ guarantees, by design, *SE(3) equivariance* with respect to its vector-valued input coordinates and features (i.e. $x_{i}\in X,\;\chi_{i}\in \chi$, and $\xi_{ij}\in \xi$) and *SE(3)-invariance* regarding its scalar features (i.e. $h_{i}\in H$ and $e_{ij}\in E$). In addition to SE(3) equivariance, Φ’s scalar graph representations achieve *geometric self-consistency* and *geometric completeness* for the 3D structure of the input molecular graph G as formalized in the definitions below, where ${□}^{\prime }$ represents an updated feature.

Definition 1 (SE(3) Equivariance).

Given $\left(H^{\prime },E^{\prime },X^{\prime },\chi^{\prime },\xi^{\prime })=\Phi (H,E,X,\chi ,\xi \right)$, we have $\left(H^{\prime },E^{\prime },QX^{\prime^{T}}+g,Q\chi^{\prime^{T}},Q\xi^{\prime^{T}})=\Phi (H,E,QX^{T}+g,Q\chi^{T},Q\xi^{T}\right)$, $\;\forall Q\in SO(3),\forall g\in R^{3\times 1}$.

Definition 2 (Geometric Self-Consistency).

Given a pair of molecular graphs $G_{1}$ and $G_{2}$, with $X^{1}={\left\{x_{i}^{1}\right\}}_{i=1,\ldots ,N}$ and $X^{2}={\left\{x_{i}^{2}\right\}}_{i=1,\ldots ,N}$, respectively, a geometric representation Φ(H,E)=Φ(G) is considered geometrically self-consistent if $\Phi (G^{1})=\Phi (G^{2})\Leftrightarrow \exists Q\in SO(3),\exists g\in R^{3\times 1}$, for $i=1,\ldots ,n,X_{i}^{1^{T}}=QX_{i}^{2^{T}}+g$ (Wang *et al.* 2022).

Definition 3 (Geometric Completeness).

Given a positional pair of nodes $\left(x_{i}^{t},x_{j}^{t}\right)$ in a 3D graph G, with vectors $a_{ij}^{t}\in R^{1\times 3},\;b_{ij}^{t}\in R^{1\times 3}$, and $c_{ij}^{t}\in R^{1\times 3}$ derived from $\left(x_{i}^{t},x_{j}^{t}\right)$, a local geometric representation ${ℱ}_{ij}^{t}=(a_{ij}^{t},b_{ij}^{t},c_{ij}^{t})\in R^{3\times 3}$ is considered geometrically complete if ${ℱ}_{ij}^{t}$ is non-degenerate, thereby forming a *local orthonormal basis* located at the tangent space of $x_{i}^{t}$Du *et al.* (2022).

##### 2.1.1.1 GCPNet model architecture

**Figure 1.**
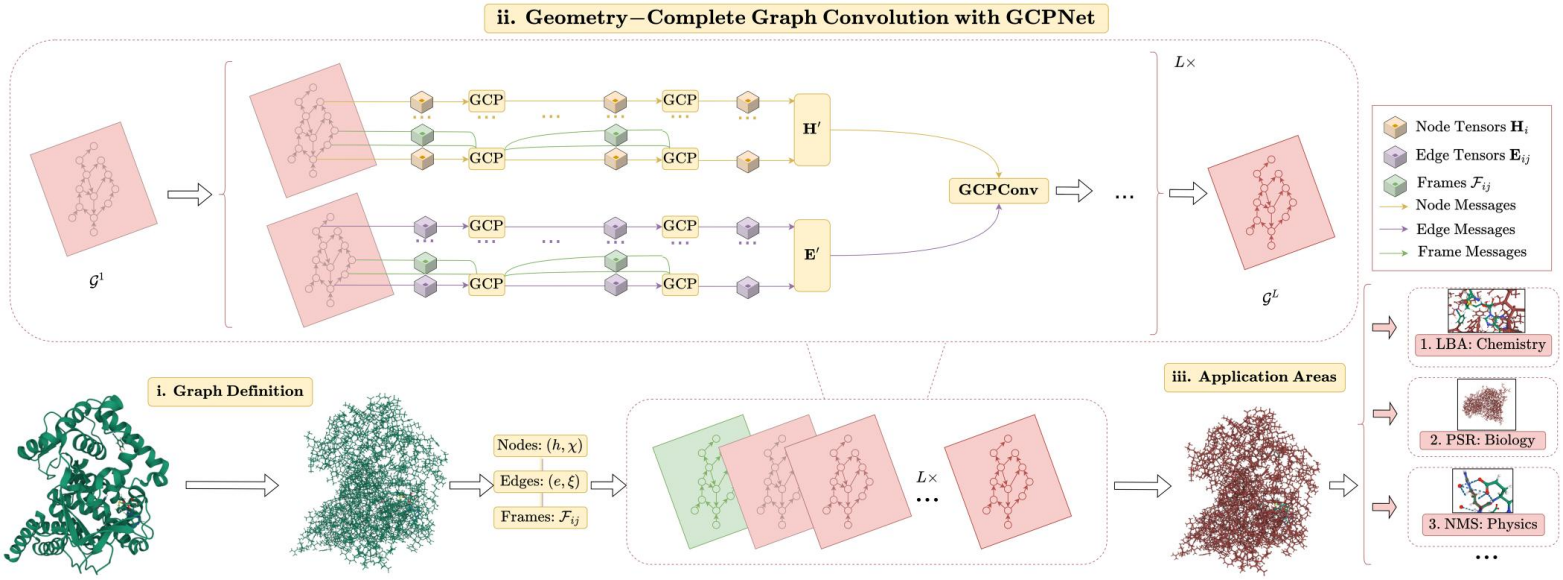
A framework overview for our proposed *Geometry-Complete Perceptron Network* (GCPNet). Our framework consists of (i) a graph (topology) definition process, (ii) a GCPNet-based graph neural network for 3D molecular representation learning, and (iii) demonstrated application areas for GCPNet. Zoom in for the best viewing experience.

**Figure 2.**
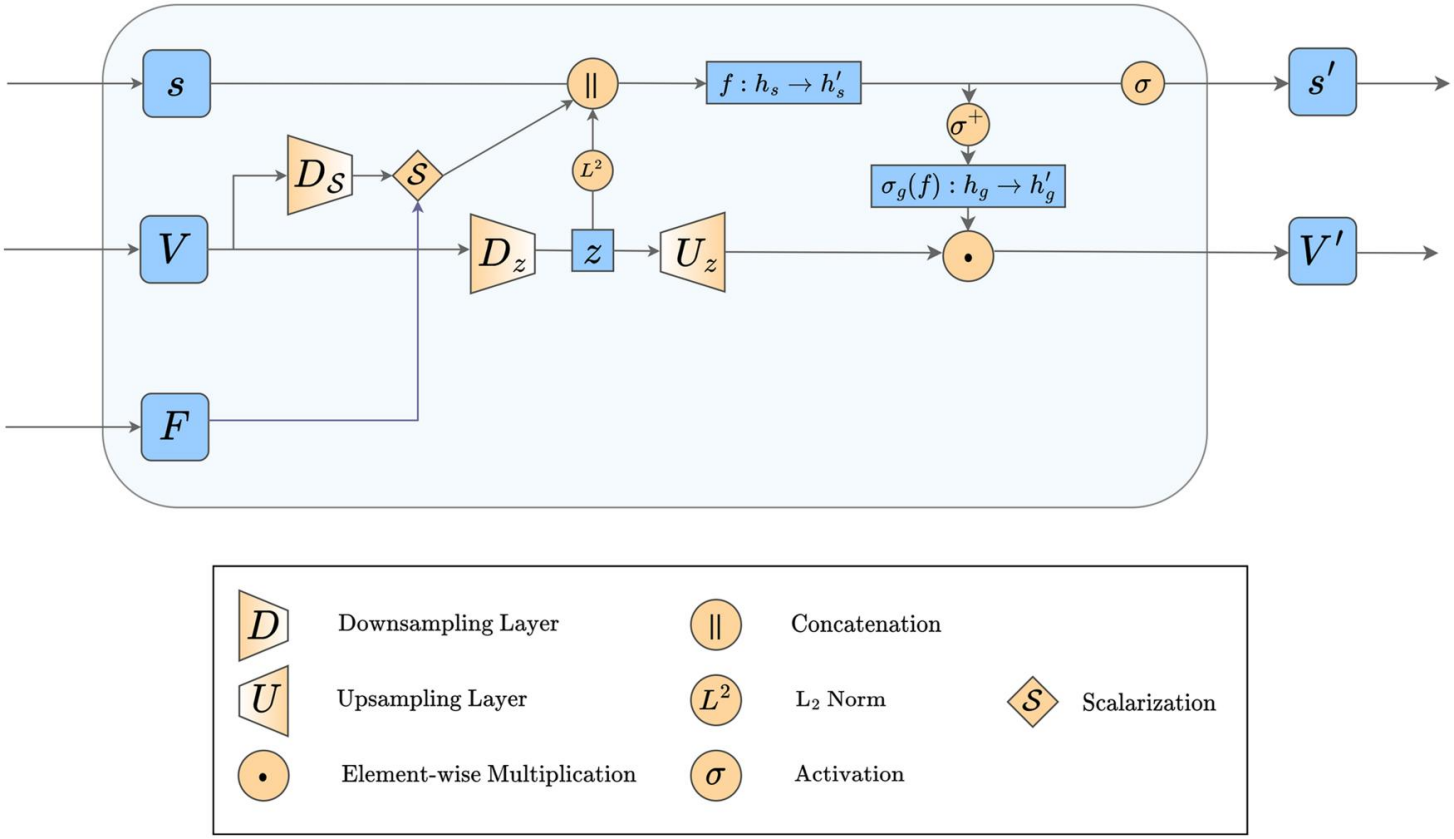
An overview of our proposed Geometry-Complete Perceptron (GCP) module. The GCP module introduces node and edge-centric encodings of 3D frames as input features that are used to directly update both scalar and vector-valued features with geometric information-completeness guarantees as well as chirality sensitivity.

To satisfy the geometric constraints described in Section 2.1.1, we introduce our architecture for Φ satisfying Definitions (1), (2), and (3) which we refer to as the Geometry-Complete SE(3)-Equivariant Perceptron Network (GCPNet). We illustrate the GCPN*et al*gorithm in Fig. 1 and outline it in Algorithm 1. Subsequently, we expand on our definition for **GCP** and **GCPConv** in Section in the main text and Supplementary Appendix A, respectively, while further illustrating **GCP** in Fig. 2.

We can then prove the following three propositions (see Supplementary Appendix B for a more detailed description of the GCPN*et al*gorithm and its corresponding property proofs).

Proposition 1. *GCPNets are SE(3)-equivariant*

→*Def. (1).*

Proposition 2. *GCPNets are geometry self-consistent*

→*Def. (2).*

Proposition 3. *GCPNets are geometry-complete*

→*Def. (3).*

#### 2.1.2 Geometry-Complete perceptron module

As illustrated in Fig. 2, GCPNet represents the features for nodes within an input graph as a tuple (h,χ) to distinguish scalar features $\left(h\in R^{h}\right)$ from vector-valued features $\left(\chi \in R^{m\times 3}\right)$. Similarly, GCPNet represents an input graph’s edge features as a tuple (e,ξ) to differentiate scalar features $\left(e\in R^{e}\right)$ from vector-valued features $\left(\xi \in R^{x\times 3}\right)$. For conciseness, we will subsequently refer to both node and edge feature tuples as (*s*, *V*). We then define $GCP_{F_{ij},\lambda }(\cdot )$ to represent the **GCP** encoding process, where *λ* represents a downscaling hyperparameter (e.g. 3) and ${ℱ}_{ij}\in R^{3\times 3}$ denotes the SO(3)-equivariant (i.e. 3D rotation-equivariant) frames constructed using the **Localize** operation (i.e. the **EquiFrame** operation of Du *et al.* (2022)) in Algorithm 1. Specifically, the frame encodings are defined as ${ℱ}_{ij}^{t}=(a_{ij}^{t},b_{ij}^{t},c_{ij}^{t})$, with $a_{ij}^{t}=\frac{x_{i}^{t}-x_{j}^{t}}{\left||x_{i}^{t}-x_{j}^{t}|\right|},b_{ij}^{t}=\frac{x_{i}^{t}\times x_{j}^{t}}{\left||x_{i}^{t}\times x_{j}^{t}|\right|},$ and $c_{ij}^{t}=a_{ij}^{t}\times b_{ij}^{t}$, respectively. In Supplementary Appendix B, we discuss how these frame encodings are direction information-complete for edges, allowing networks incorporating them to effectively detect and leverage for downstream tasks the force fields present within real-world many-body systems such as small molecules and proteins.

*Expressing Vector Representations with V.* The **GCP** module then expresses vector representations *V* as follows. The features *V* with representation depth *r* are downscaled by *λ*.

(1) $$z=\{vw_{d_{z}}|w_{d_{z}}\in R^{r\times (r/\lambda )}\}$$

Additionally, *V* is separately downscaled in preparation to be subsequently embedded as direction-sensitive edge scalar features.

(2) $$V_{s}=\{vw_{d_{s}}|w_{d_{s}}\in R^{r\times (3\times 3)}\}$$

*Deriving Scalar Representations* $s^{\prime }$. To update scalar representations, the **GCP** module, in the following manner, derives two invariant sources of information from *V* and combines them with *s*:

(3) $$q_{ij}=(V_{s}\cdot F_{ij})\in R^{9}$$

(4) $$q=\{\begin{array}{cc} \frac{1}{\left|N(i)\right|}\sum_{j\in N(i)}q_{ij} & \text{if}\;V_{s}\;\text{represents nodes}\\ q_{ij} & \text{if}\;V_{s}\;\text{represents edges} \end{array}$$

(5) $$s_{\left(s,q,z\right)}=s\cup q\cup ||z|{|}_{2}$$

where · denotes the inner product, N(·) represents the neighbors of a node, and $||\cdot |{|}_{2}$ denotes the *L*_2_ norm. Then, denote *t* as the representation depth of *s*, and let $s_{\left(s,q,z\right)}\in R^{t+9+(r/\lambda )}$ with representation depth (t+9+(r/λ)) be projected to $s^{\prime }$ with representation depth $t^{\prime }$:

(6) $$s_{v}=\{s_{\left(s,q,z\right)}w_{s}+b_{s}|w_{s}\in R^{(t+9+(r/\lambda ))\times t^{\prime }}\}$$

(7) $$s^{\prime }=\sigma_{s}(s_{v})$$

Note that embedding geometric frames $F_{ij}$ as *q_ij_* in Equation (3) ultimately enables GCPNet to iteratively learn chirality-sensitive and global force-aware representations of each 3D network input. Moreover, Equation (4) allows GCPNet to encode local geometric substructures for each node, where the theoretical importance of such network behavior is discussed in detail by Du *et al.* (2022).

*Deriving Vector Representations* $V^{\prime }$. The **GCP** module then concludes by updating vector representations as follows:

(8) $$V_{u}=\{zw_{u_{z}}|w_{u_{z}}\in R^{(r/\lambda )\times r^{\prime }}\}$$

(9) $$V^{\prime }=\{V_{u}⊙\sigma_{g}(\sigma^{+}(s_{v})w_{g}+b_{g})|w_{g}\in R^{t^{\prime }\times r^{\prime }}\}$$

where ⊙ represents element-wise multiplication and the gating function *σ_g_* is applied row-wise to preserve SO(3) equivariance within $V^{\prime }$.

Conceptually, the **GCP** module is autoregressively applied to tuples (*s*, *V*) a total of *ω* times to derive rich scalar and vector-valued features. The module does so by blending both feature types iteratively with the 3D direction and information completeness guarantees provided by geometric frame encodings ${ℱ}_{ij}$. We note that this model design runs in contrast with prior GNNs for physical systems such as GVP-GNNs (Jing *et al.* 2020, 2021) and ClofNet (Du *et al.* 2022), which are either insensitive to chemical chirality and global atomic forces or do not directly learn geometric features for downstream prediction tasks, making the proposed **GCP** module well suited for learning directly from 3D molecular graphs.

### 2.2 Learning from 3D Graphs with GCPNet

In this section, we propose a flexible manner in which to perform 3D graph convolution with our proposed **GCP** module, as illustrated in Fig. 1 and employed in Algorithm 1. For interested readers, in Supplementary Appendix B, we provide an expanded derivation and description of how to perform 3D graph convolution with GCPNet.

#### 2.2.1 The GCPN*et al*gorithm

Algorithm 1. GCPNet

**Require:** $(h_{i}\in H,\;\chi_{i}\in \chi ),\;(e_{ij}\in E,\;\xi_{ij}\in \xi ),\;x_{i}\in X$ , graph G

1: Initialize $X^{0}=X^{C}\leftarrow \mathrm{Centralize}(X)$

2: ${ℱ}_{ij}=\mathrm{Localize}(x_{i}\in X^{0},x_{j}\in X^{0})$

3: Project $\left(h_{i}^{0},\;\chi_{i}^{0}),\;(e_{ij}^{0},\;\xi_{ij}^{0})\leftarrow GCP_{e}((h_{i},\;\chi_{i}),\;(e_{ij},\;\xi_{ij}),\;F_{ij}\right)$

4: **for** *l *=* *1 **to** *L* **do**

5:   $\left(h_{i}^{l},\;\chi_{i}^{l}),\;x_{i}^{l}=\mathrm{GCPCon}v^{l}((h_{i}^{l-1},\;\chi_{i}^{l-1}),\;(e_{ij}^{0},\;\xi_{ij}^{0}),\;x_{i}^{l-1},\;F_{ij}\right)$

6: **end for**

7: **if** Updating Node Positions **then**

8:   ${ℱ}_{ij}^{L}=\mathrm{Localize}(x_{i}\in X^{l},\;x_{j}\in X^{l})$

9:   Finalize $\left(X^{L})\leftarrow \mathrm{Decentralize}(X^{l}\right)$

10: **else**

11:   $x_{i}^{L}=x_{i}^{0}$

12: **end if**

13: Project $\left(h_{i}^{L},\;\chi_{i}^{L}),\;(e_{ij}^{L},\;\xi_{ij}^{L})\leftarrow GCP_{p}((h_{i}^{l},\;\chi_{i}^{l}),\;(e_{ij}^{0},\;\xi_{ij}^{0}),\;F_{ij}^{L}\right)$

**Ensure:** $(h_{i}^{L},\;\chi_{i}^{L}),\;(e_{ij}^{L},\;\xi_{ij}^{L}),\;x_{i}^{L}$

In this section, we describe our overall 3D graph convolution learning algorithm driven by GCPNet (Algorithm 1). We also discuss the rationale behind our design decisions for GCPNet and provide examples of use cases in which one might apply GCPNet for specific learning tasks.

On Line 2 of Algorithm 1, the **Centralize** operation removes the center of mass from each node position in the input graph to ensure that such positions are subsequently 3D translation-invariant.

Thereafter, following Du *et al.* (2022), the **Localize** operation on Line 3 crafts translation-invariant and SO(3)-equivariant frame encodings ${ℱ}_{ij}^{t}=(a_{ij}^{t},b_{ij}^{t},c_{ij}^{t})$. As described in more detail in Supplementary Appendix B, these frame encodings are chirality-sensitive and direction information-complete for edges, imbuing networks that incorporate them with the ability to more easily detect force field interactions present in many real-world atomic systems, as we demonstrate through corresponding experiments in Section 3.

Before applying any geometry-complete graph convolution layers, on Line 4 we use $GCP_{e}$ to embed our input node and edge features into scalar and vector-valued values, respectively, while incorporating geometric frame information. Subsequently, in Lines 5–6, each layer of geometry-complete graph convolution is performed autoregressively via $G\mathrm{CPCon}v^{l}$ starting from these initial node and edge feature embeddings, all while maintaining information flow originating from the geometric frames ${ℱ}_{ij}$.

On Lines 8 through 12, we finalize our procedure with which to update in an SE(3)-equivariant manner the position of each node in an input 3D graph. In particular, we update node positions by residually adding learned vector-valued node features ($\chi_{v_{i}}^{l}$) to the node positions produced by the previous **GCPConv** layer (l−1). As shown in Supplementary Appendix B, such updates are initially SO(3)-equivariant, and on Line 10 we ensure these updates also become 3D translation-equivariant by adding back to each node position the input graph’s original center of mass via the **Decentralize** operation. In total, this procedure produces SE(3)-equivariant updates to node positions. Additionally, for models that update node positions, we note that Line 9 updates frame encodings ${ℱ}_{ij}$ using the model’s final predictions for node positions to provide more information-rich feature projections on Line 14 via $GCP_{p}$ to conclude the forward pass of GCPNet.

#### 2.2.2 Network utilities

In summary, GCPNet receives an input 3D graph G with node positions ***x***, scalar node and edge features, *h* and *e*, as well as vector-valued node and edge features, *χ* and *ξ*. The model is then capable of e.g. (1) predicting scalar node, edge, or graph-level properties while maintaining SE(3) invariance; (2) estimating vector-valued node, edge, or graph-level properties while ensuring SE(3) equivariance; or (3) updating node positions in an SE(3)-equivariant manner.

## 3 Results

In this work, we consider four distinct modeling tasks comprised of seven datasets in total, where implementation details are discussed in Supplementary Appendix D. We note that additional experiments are included in Supplementary Appendix C for interested readers.

### 3.1 Molecular chirality detection

#### 3.1.1 Assessing model sensitivity to molecular chirality

Molecular chirality is an essential geometric property of 3D molecules for models to consider when making predictions for downstream tasks. Simply put, this property describes the “handedness” of 3D molecules, in that, certain molecules cannot be geometrically superimposed upon a mirror reflection of themselves using only 3D rotation and translation operations. This subsequently poses a key challenge for machine learning models: Can such predictive models effectively sensitize their predictions to the effects of molecular chirality such that, under 3D reflections, their molecular feature representations change accordingly? To answer this question using modern machine learning methods, we adopt the rectus/sinister (RS) 3D molecular dataset of Adams *et al.* (2021) (i.e. a 70/15/15 train/validation/test split of PubChem3D (Bolton *et al.* 2011) where conformers correspond to the same 2D graphs in the same partition to prevent data leakage between splits) to evaluate the ability of state-of-the-art machine learning methods to distinguish between right-handed and left-handed versions of a 3D molecule. In addition, we carefully follow their experimental setup including dataset splitting; evaluation criteria; scalar feature sets of atom types, degrees, charges, numbers of hydrogens, hybridizations, and bond types and distances; and vector feature sets of atom orientations and pairwise bond displacements, respectively), where we evaluate each method’s classification accuracy in distinguishing between right and left-handed versions of a molecule. Baseline methods for this task include state-of-the-art invariant neural networks (INNs) and equivariant neural networks (ENNs), where we list each method’s latest results for this task as reported in Schneuing *et al.* (2022).

#### 3.1.2 Contribution of frame embeddings for chirality sensitivity

Table 1 shows that GCPNet is more accurately able to detect the effects of molecular chirality compared to all other baseline methods (including all other SE(3)-equivariant models), even without performing any hyperparameter tuning. In particular, GCPNet outperforms ChIRo (Adams *et al.* 2021), a GNN specifically designed to detect different forms of chirality in 3D molecules. Moreover, when we ablate GCPNet’s embeddings of local geometric frames, we find that this E(3)-equivariant (i.e. scalar-wise 3D rotation *and* reflection-invariant) version of GCPNet is no longer able to solve this important molecular recognition task, resulting in prediction accuracies at parity with random guessing. These two previous observations highlight that (1) GCPNet’s local frame embeddings are critical components of the model’s sensitivity to molecular chirality and that, (2) using such frame embeddings, GCPNet can flexibly learn representations of 3D molecules that are more predictive of chemical chirality compared to hand-crafted methods for such tasks. Moreover, these results highlight that, in order to effectively account for the effects of chirality on molecular structures, a method must be SE(3)-equivariant such that it employs SE(3)-invariant (and, thereby, reflection-varying) features for its scalar downstream predictions.

**Table 1.** Comparison of GCPNet with baseline methods for the RS task.

Type	Method	Symmetries	R/S Accuracy (%) ↑
INN	ChIRo (Schneuing *et al*. 2022)	SE(3)	98.5
	SchNet (Schneuing *et al.* 2022)	E(3)	54.4
	DimeNet++ (Schneuing *et al.* 2022)	E(3)	65.7
	SphereNet (Schneuing *et al.* 2022)	SE(3)	98.2
ENN	EGNN (Schneuing *et al.* 2022)	E(3)	50.4
	SEGNN (Schneuing *et al.* 2022)	SE(3)	83.4
Ours	GCPNet w/o Frames	E(3)	50.2 ± 0.6
	GCPNet	SE(3)	**98.7 **±** 0.1**

The results are averaged over three independent runs. The top-1 (best) results for this task are in bold, and the second-best results are underlined.

### 3.2 Protein-Ligand binding affinity prediction

#### 3.2.1 Evaluating predictions of protein–ligand binding affinity

Protein–LBA prediction challenges methods to estimate the binding affinity of a protein–ligand complex as a single scalar value (Townshend *et al.* 2020). Accurately estimating such values in a matter of seconds using a machine learning model can provide invaluable and timely information in the typical drug discovery pipeline (Rezaei *et al.* 2020). The corresponding dataset for this SE(3)-invariant task is derived from the ATOM3D dataset (Townshend *et al.* 2020) and is comprised of 4463 nonredundant protein–ligand complexes, where cross-validation splits are derived using a strict 30% sequence identity cutoff. Results are reported in terms of the root mean squared error (RMSE), Pearson’s correlation (*p*), and Spearman’s correlation (*Sp*) between a method’s predictions on the test dataset and the corresponding ground-truth binding affinity values represented as $pK=-{\;\text{log}\;}_{10}(K)$, where *K* is the binding affinity measured in Molar units. Baseline comparison methods for this task include a variety of state-of-the-art CNNs, recurrent neural networks (RNNs), GNNs, and ENNs, with additional baselines utilizing explicit protein-ligand interaction information listed in Supplementary Table S2. Using the same dataset and dataset splits, results for these methods are reported as in Wang *et al.* (2023b), Aykent and Xia (2022), and Liu *et al.* (2023), respectively. Note, however, that due to their lack of official publicly-available PyTorch Geometric (Fey and Lenssen 2019) source code, for this task we include simple PyTorch Geometric reproductions of PaiNN (Schütt *et al.* 2021) and the Equivariant Transformer (ET) (Thölke and De Fabritiis 2022) as additional equivariant GNN and Transformer baselines, respectively. Consequently, due to computational resource constraints, we do not perform any hyperparameter tuning for these two methods.

The results shown in Table 2 reveal that, in operating on atom-level protein-ligand graph representations, GCPNet achieves the best performance for predicting protein–LBA by a significant margin, notably improving performance across all metrics by 7% on average. Here, to the best of our knowledge, GCPNet is one of the first methods capable of achieving Pearson and Spearman binding affinity correlations greater than 0.6 on the PDBBind dataset (Wang *et al.* 2005) curated as part of the ATOM3D benchmark (which employs a strict 30% sequence identity cutoff) (Townshend *et al.* 2020). Moreover, we find that these correlations are highly statistically significant (i.e. Pearson’s *P*-value of 2e−50, Spearman’s *P*-value of 2e−49, and Kendall’s tau correlation of 0.432 with a *P*-value of 3e−45).

**Table 2.** Comparison of GCPNet with baseline methods for the LBA task.

Type	Method	RMSE ↓	*p* ↑	*Sp* ↑
CNN	3DCNN (Wang *et al.* 2023b)	1.416 ± 0.021	0.550	0.553
	DeepDTA (Wang *et al.* 2023b)	1.866 ± 0.080	0.472	0.471
	DeepAffinity (Aykent and Xia 2022)	1.893 ± 0.650	0.415	0.426
RNN	Bepler and Berger (Wang *et al.* 2023b)	1.985 ± 0.006	0.165	0.152
	TAPE (Wang *et al.* 2023b)	1.890 ± 0.035	0.338	0.286
	ProtTrans (Wang *et al.* 2023b)	1.544 ± 0.015	0.438	0.434
GNN	GCN (Wang *et al.* 2023b)	1.601 ± 0.048	0.545	0.533
	DGAT (Aykent and Xia 2022)	1.719 ± 0.047	0.464	0.472
	DGIN (Aykent and Xia 2022)	1.765 ± 0.076	0.426	0.432
	DGAT-GCN (Aykent and Xia 2022)	1.550 ± 0.017	0.498	0.496
	MaSIF (Wang *et al.* 2023b)	1.484 ± 0.018	0.467	0.455
	IEConv (Wang *et al.* 2023b)	1.554 ± 0.016	0.414	0.428
	Holoprot-Full Surface (Wang *et al.* 2023b)	1.464 ± 0.006	0.509	0.500
	Holoprot-Superpixel (Wang *et al.* 2023b)	1.491 ± 0.004	0.491	0.482
	ProNet-Amino-Acid (Wang *et al.* 2023b)	1.455 ± 0.009	0.536	0.526
	ProNet-Backbone (Wang *et al.* 2023b)	1.458 ± 0.003	0.546	0.550
	ProNet-All-Atom (Wang *et al.* 2023b)	1.463 ± 0.001	0.551	0.551
	GeoSSL-DDM (Liu *et al.* 2023)	1.451 ± 0.030	0.577	0.572
ENN	Cormorant (Aykent and Xia 2022)	1.568 ± 0.012	0.389	0.408
	PaiNN	1.698 ± 0.050	0.366	0.358
	ET	1.490 ± 0.019	0.564	0.532
	GVP (Aykent and Xia 2022)	1.594 ± 0.073	0.434	0.432
	GBP (Aykent and Xia 2022)	1.405 ± 0.009	0.561	0.557
Ours	GCPNet w/o Frames	1.485 ± 0.015	0.521	0.504
	GCPNet w/o ResGCP	1.514 ± 0.008	0.471	0.468
	GCPNet w/o Scalars	1.685 ± 0.000	0.050	0.000
	GCPNet w/o Vectors	1.727 ± 0.005	0.270	0.304
	GCPNet	**1.352 **±** 0.003**	**0.608**	**0.607**

The results are averaged over three independent runs. The top-1 (best) results for this task are in bold, and the second-best results are underlined.

#### 3.2.2 Ablating network components reveals impact of model design

Denoted as “GCPNet w/o …” in Table 2, our ablation studies with GCPNet for the LBA task demonstrate the contribution of each component in its model design. In particular, our proposed local frame embeddings improve GCPNet’s performance by more than 15% across all metrics (GCPNet w/o Frames), where we hypothesize these performance improvements come from using these frame embeddings to enhance the model’s sensitivity to molecular chirality. Similarly, our proposed residual GCP module (i.e. ResGCP) improves GCPNet’s performance by 23% on average.

Specifically of interest is the observation that independent removal of scalar and vector-valued features within GCPNet appears to severely decrease GCPNet’s performance for LBA prediction. Notably, removing the model’s access to scalar-valued node and edge features (i.e. one-hot atom types and edge distance embeddings, respectively) degrades performance by 70% on average, while not allowing the model to access vector-valued node and edge features (i.e. sequence-based orientation vectors and pairwise atom displacement vectors, respectively) reduces performance by 42% on average. One possible explanation for these observations is that both types of feature representations the baseline GCPNet model learns (i.e. scalars and vectors) are useful for understanding protein-ligand interactions. In addition, our ablation results in Table 2 suggest that our proposed frame embeddings and ResGCP module are complementary to these scalar and vector-valued features in the context of predicting the binding affinity of a protein-ligand complex.

### 3.3 Protein model quality assessment

#### 3.3.1 Evaluating ranking predictions for protein structure decoys

Protein structure ranking requires methods to predict the overall quality of a 3D protein structure when comparing it to a reference (i.e. native) protein structure (Townshend *et al.* 2020). The quality of a protein structure is reported as a single scalar value representing a method’s predicted global distance test (GDT_TS) score (Zemla 2003) between the provided decoy structure and the native structure. Such information is crucial in drug discovery efforts when one is tasked with designing a drug (e.g. ligand) that should bind to a particular protein target, notably when such targets have not yet had their 3D structures experimentally determined and have rather had them predicted computationally using methods such as AlphaFold 2 (Jumper *et al.* 2021). The respective dataset for this SE(3)-invariant task is also derived from the ATOM3D dataset (Townshend *et al.* 2020) and is comprised of 40 950 decoy structures corresponding to 649 total targets, where cross-validation splits are created according to a target’s release year in the Critical Assessment of Techniques for Protein Structure Prediction (CASP) competition (Kryshtafovych *et al.* 2021). Results are reported in terms of the Pearson’s correlation (*p*), Spearman’s correlation (*Sp*), and Kendall’s tau correlation (*K*) between a method’s predictions on the test dataset and the corresponding ground-truth GDT_TS values, where local results are averaged across predictions for individual targets and global results are averaged directly across all targets. Baseline comparison methods for this task include a composition of state-of-the-art CNNs, GNNs, and ENNs (including our reproductions of PaiNN and ET), as well as previous statistics-based methods. Using the same dataset and dataset splits, results for these methods are reported as in Aykent and Xia (2022) and Townshend *et al.* (2020), respectively.

Conveying a similar message to that in Table 2, the results in Table 3 demonstrate that, in operating on atom-level protein graphs, GCPNet performs best against all other state-of-the-art models for the task of estimating a 3D protein structure’s quality (i.e. PSR). In this setting, GCPNet outperforms all other methods across all local and global metrics by 2.5% on average. Once again, GCPNet’s predictions are highly statistically significant, this time with Pearson, Spearman, and Kendall tau *P*-values all below 1e−50, respectively.

**Table 3.** Comparison of GCPNet with baseline methods for the PSR task.

	Local			Global		
Method	*p* ↑	*Sp* ↑	*K* ↑	*p* ↑	*Sp* ↑	*K* ↑
3DCNN (Aykent and Xia 2022)	0.557	0.431	0.308	0.780	0.789	0.592
GCN (Townshend *et al.* 2020)	0.500	0.411	0.289	0.747	0.750	0.547
ProQ3D (Aykent and Xia 2022)	0.444	0.432	0.304	0.796	0.772	0.594
VoroMQA (Aykent and Xia 2022)	0.412	0.419	0.291	0.688	0.651	0.505
RWplus (Aykent and Xia 2022)	0.192	0.167	0.137	0.033	0.056	0.011
SBROD (Aykent and Xia 2022)	0.431	0.413	0.291	0.551	0.569	0.393
Ornate (Aykent and Xia 2022)	0.393	0.371	0.256	0.625	0.669	0.481
DimeNet (Aykent and Xia 2022)	0.302	0.351	0.285	0.614	0.625	0.431
GraphQA (Aykent and Xia 2022)	0.357	0.379	0.251	0.821	0.820	0.618
PaiNN	0.518	0.444	0.315	0.773	0.813	0.611
ET	0.564	0.466	0.330	0.813	0.814	0.611
GVP (Aykent and Xia 2022)	0.581	0.462	0.331	0.805	0.811	0.616
GBP (Aykent and Xia 2022)	0.612	0.517	0.372	0.856	0.853	0.656
GCPNet w/o Frames	0.588	0.512	0.367	0.854	0.851	0.657
GCPNet w/o ResGCP	0.576	0.509	0.365	0.852	0.847	0.648
GCPNet w/o Scalars	N/A	N/A	N/A	N/A	N/A	N/A
GCPNet w/o Vectors	0.571	0.497	0.356	0.802	0.804	0.608
GCPNet	**0.616**	**0.534**	**0.385**	**0.871**	**0.869**	**0.676**

Local metrics are averaged across target-aggregated metrics. The best results for this task are in bold, and the second-best results are underlined. N/A denotes a metric that could not be computed.

#### 3.3.2 Identifying components for effective protein structure ranking

Our ablation studies with GCPNet, in the context of PSR, once more reveal that the design of our local frames, ResGCP module, and scalar and vector feature channels are all beneficial for enhancing GCPNet’s ability to analyze a given 3D graph input. Here, in sensitizing the model to chemical chirality, our local frame embeddings improve GCPNet’s performance for PSR by 4% on average. Similarly, our ResGCP module improves the model’s performance by 5%. Interestingly, without access to scalar-valued node and edge features (i.e. the same as those used for the LBA task), GCPNet is unable to produce valid predictions for the PSR test dataset due to what appears to be a phenomenon of vector-wise latent variable collapse (Dieng *et al.* 2019). This finding suggests that, for the PSR task, the baseline GCPNet model relies strongly on the scalar-valued representations it produces. Lastly, including vector-valued node and edge features (i.e. the same as those used for the LBA task) within GCPNet improves the model’s performance for the PSR task by 9%.

### 3.4 Future position forecasting for newtonian particle systems

#### 3.4.1 Evaluating trajectory predictions for Newtonian many-body systems

Newtonian many-body systems modeling (NMS) asks methods to forecast the future positions of particles in many-body systems of various sizes (Du *et al.* 2022), bridging the gap between the domains of machine learning and physics. In our experimental results for the NMS task, the four systems (i.e. datasets) on which we evaluate each method are comprised of increasingly more nodes and are influenced by force fields of increasingly complex directional origins for which to model, namely electrostatic force fields for 5-body (ES(5)) and 20-body (ES(20)) systems as well as for 20-body systems under the influence of an additional gravity field (G+ES(20)) and Lorentz-like force field (L+ES(20)), respectively. The four datasets for this SE(3)-equivariant task were generated using the descriptions and source code of Du *et al.* (2022), where each dataset is comprised of 7000 total trajectories. Results are reported in terms of the mean squared error (MSE) between a method’s node position predictions on the test dataset and the corresponding ground-truth node positions after 1000 timesteps. Baseline comparison methods for this task include a collection of state-of-the-art GNNs, ENNs, and Transformers (including our reproductions of PaiNN and ET), where we list each method’s latest results for this task as reported in Du *et al.* (2022).

The results in Table 4 show that GCPNet achieves the lowest MSE averaged across all four NMS datasets, improving upon the state-of-the-art MSE for trajectory predictions in this task by 19% on average. In particular, GCPNet achieves the best results for two of the four NMS datasets considered in this work, where these two datasets are respectively the first and third most difficult NMS datasets for methods to model. On the two remaining datasets, GCPNet matches the performance of prior state-of-the-art methods such as ClofNet (Du *et al.* 2022). Moreover, across all four datasets, GCPNet’s trajectory predictions yield an RMSE of 0.0963 and achieve Pearson, Spearman, and Kendall’s tau correlations of 0.999, 0.999, and 0.981, respectively, where all such correlation values are highly statistically significant (i.e. *P*-values <1e−50). Note that, to calculate these correlation values, we score GCPNet’s vector-valued predictions independently for each coordinate axis and then average the resulting metrics. Also note that we only compare methods such as ClofNet to GCPNet in the context of the NMS task, as e.g. ClofNet is specifically designed always to predict new 3D coordinates for each of its 3D graph inputs, with coordinate updates being the primary predictive target for the NMS dataset but with other tasks not targeting updated coordinates.

**Table 4.** Comparison of GCPNet with baseline methods for the NMS task.

Method	ES(5)	ES(20)	G+ES(20)	L+ES(20)	Average
GNN (Du *et al.* 2022)	0.0131	0.0720	0.0721	0.0908	0.0620
TFN (Du *et al.* 2022)	0.0236	0.0794	0.0845	0.1243	0.0780
SE(3)-Transformer (Du *et al.* 2022)	0.0329	0.1349	0.1000	0.1438	0.1029
Radial Field (Du *et al.* 2022)	0.0207	0.0377	0.0399	0.0779	0.0441
PaiNN	0.0158	N/A	N/A	N/A	N/A
ET	0.1653	0.1788	0.2122	0.2989	0.2138
EGNN (Du *et al.* 2022)	0.0079	0.0128	0.0118	0.0368	0.0173
ClofNet (Du *et al.* 2022)	**0.0065**	0.0073	**0.0072**	0.0251	0.0115
GCPNet w/o Frames	0.0067	0.0074	0.0074	0.0200	0.0103
GCPNet w/o ResGCP	0.0090	0.0135	0.0099	0.0278	0.0150
GCPNet w/o Scalars	0.0119	0.0173	0.0170	0.0437	0.0225
GCPNet	0.0070	**0.0071**	0.0073	**0.0173**	**0.0097**

Results are reported in terms of the MSE for future position prediction over four test datasets of increasing modeling difficulty, graph sizes, and composed force field complexities. The final column reports each method’s MSE averaged across all four test datasets. The best results for this task are in bold, and the second-best results are underlined. N/A denotes an experiment that could not be performed due to a method’s numerical instability.

#### 3.4.2 Analyzing components for successful trajectory forecasting

Once again, our ablation studies with GCPNet demonstrate the importance of GCPNet’s local frame embeddings, scalar node and edge features (i.e. invariant velocity encodings and edge type and distance embeddings, respectively), and ResGCP module. Here, we note that we were not able to include an ablation study on GCPNet’s vector-valued node and edge features (i.e. velocity and orientation encodings as well as pairwise atom displacements, respectively) since they are directly used to predict node position displacements for trajectory forecasting. Table 4 shows that each model component synergistically enables GCPNet to achieve new state-of-the-art results for the NMS task. In enabling the model to detect global forces, our proposed local frame embeddings improve GCPNet’s ability to learn many-body system dynamics by 6% on average across all dataset contexts. Specifically interesting to note is that these local frame embeddings improve the model’s trajectory predictions within the most complex dataset context (i.e. L+ES(20)) by 14%, suggesting that such frame embeddings improve GCPNet’s ability to learn many-body system dynamics even in the presence of complex global force fields. Furthermore, GCPNet’s ResGCP module and scalar-valued features improve the model’s performance for modeling many-body systems by 35% and 57%, respectively.

Across all tasks studied in this work, GCPNet improves upon the overall performance of all previous methods. Our experiments demonstrate this for both node-level (e.g. NMS) and graph-level (e.g. LBA) prediction tasks, verifying GCPNet’s ability to encode useful information for both scales of granularity. Furthermore, we have demonstrated the importance of each model component within GCPNet, showing how these components are complementary to each other in the context of representation learning over 3D molecular data. Lastly, in Supplementary Table S10, we report the run time of GCPNet on each task’s test dataset to enable future methods to directly compare their computational run time to that of GCPNet.

## 4 Conclusion

In this work, we introduced GCPNet, a state-of-the-art GNN for 3D molecular graph representation learning. We have demonstrated its utility through several benchmark studies. In future work, we aim to develop extensions of GCPNet that increase its geometric expressiveness as well as explore applications of GCPNet for generative modeling of molecular structures.

## Supplementary Material

btae087_Supplementary_Data

## Data Availability

The data used in this work for the NMS task are available under a CC BY 4.0 license at https://zenodo.org/record/7293186. The data used for the RS task are available under an MIT License at https://figshare.com/s/e23be65a884ce7fc8543. The remaining code and data for this work are available at https://github.com/BioinfoMachineLearning/GCPNet.

## References

[btae087-B1] Adams K , Pattanaik L, Coley C. Learning 3d representations of molecular chirality with invariance to bond rotations. In: **The Ninth* International Conference on Learning Representations,* Virtual Only, 2021*.*

[btae087-B2] Aykent S , Xia T. Gbpnet: universal geometric representation learning on protein structures. In: *Proceedings of the 28th ACM SIGKDD Conference on Knowledge Discovery and Data Mining*, KDD ’22, p. 4–14, New York, NY, USA. Association for Computing Machinery, 2022.

[btae087-B3] Baldassarre F , Menéndez Hurtado D, Elofsson A et al Graphqa: protein model quality assessment using graph convolutional networks. Bioinformatics 2021;37:360–6. 32780838 10.1093/bioinformatics/btaa714 PMC8058777

[btae087-B4] Batzner S , Musaelian A, Sun L et al E (3)-equivariant graph neural networks for data-efficient and accurate interatomic potentials. Nat Commun 2022;13:2453. 35508450 10.1038/s41467-022-29939-5 PMC9068614

[btae087-B5] Bolton EE , Chen J, Kim S et al Pubchem3d: a new resource for scientists. J Cheminform 2011;3:32–15. 21933373 10.1186/1758-2946-3-32 PMC3269824

[btae087-B6] Cohen T , Welling M. Group equivariant convolutional networks. In: *International Conference on Machine Learning*, *New York, NY, USA*, p. 2990–2999. PMLR, 2016.

[btae087-B7] Dieng AB , Kim Y, Rush AM et al Avoiding latent variable collapse with generative skip models. In: *The 22nd International Conference on Artificial Intelligence and Statistics, Naha, Okinawa, Japan*, p. 2397–2405. PMLR, 2019.

[btae087-B8] Du W , Zhang H, Du Y et al Se (3) equivariant graph neural networks with complete local frames. In: *International Conference on Machine Learning, Baltimore, MD, USA*, p. 5583–5608. PMLR, 2022.

[btae087-B9] Fey M , Lenssen JE. Fast graph representation learning with pytorch geometric. In: *ICLR Workshop on Representation Learning on Graphs and Manifolds, New Orleans, LA, USA*, 2019.

[btae087-B10] Fuchs FB , Wagstaff E, Dauparas J et al Iterative se (3)-transformers. In: *Geometric Science of Information: 5th International Conference, GSI 2021, Paris, France, July 21–23, 2021, Proceedings 5*, p. 585–595. Springer, 2021.

[btae087-B11] Jing B , Eismann S, Suriana P et al Learning from protein structure with geometric vector perceptrons. In: *The Ninth International Conference on Learning Representations, Virtual Only, 2021.*

[btae087-B12] Jing B , Eismann S, Soni PN *et al*. Equivariant graph neural networks for 3d macromolecular structure. In: *ICML Workshop on Computational Biology, Virtual Only*, 2021.

[btae087-B13] Jumper J , Evans R, Pritzel A et al Highly accurate protein structure prediction with alphafold. Nature 2021;596:583–9. 34265844 10.1038/s41586-021-03819-2 PMC8371605

[btae087-B14] Karimi M , Wu D, Wang Z et al Deepaffinity: interpretable deep learning of compound–protein affinity through unified recurrent and convolutional neural networks. Bioinformatics 2019;35:3329–38. 30768156 10.1093/bioinformatics/btz111 PMC6748780

[btae087-B15] Kryshtafovych A , Schwede T, Topf M et al Critical assessment of methods of protein structure prediction (casp)—round xiv. Proteins: Struct Funct Bioinf 2021;89:1607–17. 10.1002/prot.26237 PMC8726744 34533838

[btae087-B16] Liu S , Guo H, Tang J. Molecular geometry pretraining with SE(3)-invariant denoising distance matching. In: *The Eleventh International Conference on Learning Representations*, *Kigali, Rwanda,* 2023.

[btae087-B17] Ma H , Bian Y, Rong Y et al Cross-dependent graph neural networks for molecular property prediction. Bioinformatics 2022;38:2003–9. 35094072 10.1093/bioinformatics/btac039

[btae087-B18] Morehead A , Chen C, Cheng J. Geometric transformers for protein interface contact prediction. In: **The Tenth* International Conference on Learning Representations*, *Virtual Only,* 2022a.

[btae087-B19] Morehead A , Chen X, Wu T et al Egr: Equivariant graph refinement and assessment of 3d protein complex structures, arXiv, arXiv:2205.10390, 2022b, preprint: not peer reviewed.

[btae087-B20] Rezaei MA , Li Y, Wu D et al. Deep learning in drug design: protein-ligand binding affinity prediction. In: *IEEE/ACM Transactions on Computational Biology and Bioinformatics*, *Online Only,* 2020. 10.1109/TCBB.2020.3046945 PMC8942327 33360998

[btae087-B21] Schneuing A , Du Y, Harris C et al Structure-based drug design with equivariant diffusion models. In: *NeurIPS Workshop on Machine Learning in Structural Biology, New Orleans, LA, USA*, 2022.

[btae087-B22] Schütt K , Unke OT, Gastegger M. Equivariant message passing for the prediction of tensorial properties and molecular spectra. In: *International Conference on Machine Learning, Virtual Only*, p. 9377–9388. PMLR, 2021.

[btae087-B23] Thölke P , De Fabritiis G. Equivariant transformers for neural network based molecular potentials. In: *International Conference on Learning Representations*, *Virtual Only*, 2022.

[btae087-B24] Thomas N , Smidt T, Kearnes S et al Tensor field networks: rotation- and translation-equivariant neural networks for 3d point clouds. arXiv, abs/1802.08219, 2018.

[btae087-B25] Townshend RJ , Vögele M, Suriana P et al Atom3d: tasks on molecules in three dimensions. In: *Advances in**Neural Information Processing Systems Datasets and Benchmarks Track, Virtual Only*, 2021.

[btae087-B26] Wang K , Zhou R, Tang J et al GraphscoreDTA: optimized graph neural network for protein–ligand binding affinity prediction. Bioinformatics2023a;39:btad340. 10.1093/bioinformatics/btad340 37225408 PMC10243863

[btae087-B27] Wang L , Liu H, Lin Y et al ComENet: towards complete and efficient message passing for 3d molecular graphs. In: Advances in Neural Information Processing Systems, New Orleans, LA, USA, 2022.

[btae087-B28] Wang L , Liu H, Liu Y et al Learning hierarchical protein representations via complete 3d graph networks. In: *The Eleventh International Conference on Learning Representations*, *Kigali, Rwanda,* 2023b.

[btae087-B29] Wang R , Fang X, Lu Y et al The pdbbind database: methodologies and updates. J Med Chem 2005;48:4111–9. 15943484 10.1021/jm048957q

[btae087-B30] Wu Y , Gao M, Zeng M et al Bridgedpi: a novel graph neural network for predicting drug–protein interactions. Bioinformatics 2022;38:2571–8. 35274672 10.1093/bioinformatics/btac155

[btae087-B31] Xia T , Ku WS. Geometric graph representation learning on protein structure prediction. In: *Proceedings of the 27th ACM SIGKDD Conference on Knowledge Discovery & Data Mining*, *Virtual Only*, p. 1873–1883, 2021.

[btae087-B32] Zemla A. Lga: a method for finding 3d similarities in protein structures. Nucleic Acids Res 2003;31:3370–4. 12824330 10.1093/nar/gkg571 PMC168977

