# Supplementary material for: Geometry-complete perceptron networks for 3D molecular graphs
Source: Bioinformatics. 2024 Feb 19;40(2):btae087. doi: 10.1093/bioinformatics/btae087 (PMC10904142; doi:10.1093/bioinformatics/btae087)
Supplement: btae087_Supplementary_Data [file btae087_supplementary_data.pdf]

Bioinformatics, 2022, 1–9
doi: DOI HERE
Advance Access Publication Date: Day Month Year
Paper

Supplementary Material for "Geometry-Complete Perceptron Networks for 3D Molecular Graphs"

Alex Morehead[1,*] and Jianlin Cheng[1]

[1]Electrical Engineering & Computer Science, University of Missouri-Columbia, W1024 Lafferre Hall, 65211, Columbia, Missouri, United States of America

*Corresponding author. acmwhb@missouri.edu

FOR PUBLISHER ONLY Received on Date Month Year; revised on Date Month Year; accepted on Date Month Year

Abstract

Motivation. The field of geometric deep learning has recently had a profound impact on several scientific domains such as protein structure prediction and design, leading to methodological advancements within and outside of the realm of traditional machine learning. Within this spirit, in this work, we introduce GCPNet, a new chirality-aware SE(3)-equivariant graph neural network designed for representation learning of 3D biomolecular graphs. We show that GCPNet, unlike previous representation learning methods for 3D biomolecules, is widely applicable to a variety of invariant or equivariant node-level, edge-level, and graph-level tasks on biomolecular structures while being able to (1) learn important chiral properties of 3D molecules and (2) detect external force fields.
Results. Across four distinct molecular-geometric tasks, we demonstrate that GCPNet's predictions (1) for protein-ligand binding affinity achieve a statistically significant correlation of 0.608, more than 5% greater than current state-of-the-art methods; (2) for protein structure ranking achieve statistically significant target-local and dataset-global correlations of 0.616 and 0.871, respectively; (3) for Newtownian many-body systems modeling achieve a task-averaged mean squared error less than 0.01, more than 15% better than current methods; and (4) for molecular chirality recognition achieve a state-of-the-art prediction accuracy of 98.7%, better than any other machine learning method to date.
Availability and Implementation. The source code, data, and instructions to train new models or reproduce our results are freely available at https://github.com/BioinfoMachineLearning/GCPNet.
Contact. acmwhb@missouri.edu

Key words: Geometric deep learning, Equivariance, Graph neural networks, Chirality, Biomolecular structure

A. Expanded Methodology Discussion

As a continuation of our methodological overview of GCPNet given in the main text in Section 1.1.1, here we further describe the equivariance, geometric self-consistency, and geometric completeness constraints that GCPNet satisfies.

As discussed in Section 1.1.1 of the main text, our GCPNet function $\mathbf{\Phi}$ guarantees, by design, *SE(3) equivariance* with respect to its vector-valued input coordinates and features (i.e., $x_i \in \mathbf{X}$, $\chi_i \in \boldsymbol{\chi}$, and $\xi_{ij} \in \boldsymbol{\xi}$) and *SE(3)-invariance* regarding its scalar features (i.e., $h_i \in \mathbf{H}$ and $e_{ij} \in \mathbf{E}$). In addition, $\mathbf{\Phi}$'s scalar graph representations achieve *geometric self-consistency* for the 3D structure of the input molecular graph $\mathcal{G}$, sensitizing them to the effects of molecular chirality while making them uniquely identifiable under 3D rotations. Lastly, geometric completeness requires methods that accept 3D molecular graph inputs to be able to discern the local geometric environment of a given atom with no directional ambiguities. This enables geometry-complete methods such as $\mathbf{\Phi}$ to detect the presence and influence of global force fields acting on the graph inputs. Note that we more carefully formalize these equivariance, geometric self-consistency, and geometric completeness constraints using three corresponding definitions in Section 1.1.1 of the main text.

SE(3)-equivariant complete representations

As described in the three definitions referenced above, representation learning on 3D molecular structures is a challenging task for a variety of reasons: (1) an expressive representation learning model should be able to predict arbitrary vector-valued quantities for each atom and atom pair in the molecular structure (e.g., using $\boldsymbol{\chi}'$ and $\boldsymbol{\xi}'$ to predict side-chain atom positions and atom-atom displacements for each residue in a 3D protein graph); (2) arbitrary rotations or translations to a 3D molecular structure

© The Author 2022. Published by Oxford University Press. All rights reserved. For permissions, please e-mail: journals.permissions@oup.com

should affect only the vector-valued representations a model assigns to a molecular graph's nodes or edges, whereas such 3D transformations of the molecular structure should not affect the model's scalar representations for nodes and edges (Du et al., 2022); (3) the geometrically invariant properties of a molecule's 3D structure should be uniquely identifiable by a model; and (4) in a geometry-complete manner, scalar and vector-valued representations should mutually exchange information between nodes and edges during a model's forward pass for a 3D input graph, as these information types can be correlatively related (e.g., a scalar feature such as the L_2 norm of a vector v can be associated with the vector of origin v) (Aykent and Xia, 2022; Morehead et al., 2022).

In line with this reasoning, we need to ensure that the coordinates our model predicts for the node positions in a molecular graph $\mathcal{G}$ transform according to SE(3) transformations of the input positions. This runs in contrast to previous methods that remain strictly E(3)-equivariant or E(3)-invariant to 3D transformations of the input $\mathcal{G}$ and consequently ignore the important effects of molecular chirality. At the same time, the model should jointly update the scalar and vector-valued features of $\mathcal{G}$ according to their respective molecular symmetry groups to increase the model's expressiveness in approximating geometric and physical quantities (Brandstetter et al., 2021). To increase its generalization capabilities, the model should also disambiguate any *geometric directions* within its local node environments and should maintain SE(3)-invariance of its scalar representations when the input graph is transformed in 3D space. Following Wang et al. (2023b), this helps prevent the model from losing important geometric or chiral information (i.e., becoming geometrically self-inconsistent) during graph message-passing. One way to do this is to introduce a new type of message-passing neural network such as GCPNet, as we have proposed in this work.

Geometry-Complete Graph Convolution with GCPNet.

As a continuation of Section 1.3 in the main text in which we will now give a more detailed derivation of how one can perform 3D graph convolution using GCPNet, let $\mathcal{N}(\mathrm{i})$ denote the neighbors of node n_i, selected using a distance-based metric such as k-nearest neighbors or a radial distance cutoff. Subsequently, we define a single layer l of geometry-complete graph convolution as

$$n_i^l = \phi^l(n_i^{l-1}, \mathcal{A}_{\forall j \in \mathcal{N}(i)} \Omega_\omega^l(n_i^{l-1}, n_j^{l-1}, e_{ij}, \mathcal{F}_{ij})), \quad (1)$$

where $n_i^l = (h_i^l, \chi_i^l)$; $e_{ij} = (e_{ij}^0, \xi_{ij}^0)$; $\mathbf{\Phi}$ is a trainable function denoted as **GCPConv**; l signifies the representation depth of the network; $\mathcal{A}$ is a permutation-invariant aggregation function; and Ω_ω represents a message-passing function corresponding to the ω-th **GCP** message-passing layer. We proceed to expand on the operations of each graph convolution layer as follows.

To start, messages between source nodes i and neighboring nodes j are first constructed as

$$m_{ij}^0 = \mathbf{GCP}(n_i^0 \cup n_j^0 \cup e_{ij}, \mathcal{F}_{ij}) \quad (2)$$

where $\cup$ denotes a concatenation operation. Then, up to the ω-th iteration, each message is updated by the m-th message update layer using residual connections as

$$\Omega_\omega^l = \mathbf{ResGCP}_\omega^l(m_{ij}^{l-1}, \mathcal{F}_{ij}), \quad (3)$$

$$\mathbf{ResGCP}_\eta^l(z_i^{l-1}, \mathcal{F}_{ij}) = z_i^{l-1} + \mathbf{GCP}_\eta^l(z_i^{l-1}, \mathcal{F}_{ij}), \quad (4)$$

where we empirically find such residual connections between message representations to reduce oversmoothing within GCPNet by mitigating the problem of vanishing gradients.

Updated node features $\hat{n}^l$ are then derived residually using an aggregation of generated messages as

$$\hat{n}^l = n^{l-1} + f(\{\Omega_{\omega,v_i}^l | v_i \in \mathcal{V}\}), \quad (5)$$

where f represents an aggregation function such as a summation or mean that is invariant to permutations of node ordering. The residual connection between $\hat{n}^l$ and n^l is established here to encourage the network to update the representation space of node features in a layer-asynchronous manner.

To encourage GCPNet to make its node feature representations independent of the size of each input graph, we then employ a node-centric feed-forward network to update node representations. Specifically, we apply to $\hat{n}^l$ a linear **GCP** function with shared weights ϕ_f followed by r **ResGCP** modules, operations concisely portrayed as

$$\tilde{n}_{r-1}^l = \phi_f^l(\hat{n}^l) \quad (6)$$

$$n^l = \mathbf{ResGCP}_r^l(\tilde{n}_{r-1}^l). \quad (7)$$

Lastly, if one desires to update the positions of each node in $\mathcal{G}$ (e.g., as we do for tasks involving position-related predictions such as NMS), we propose a flexible, SE(3)-equivariant method to do so using a dedicated **GCP** module as follows:

$$(h_{p_i}^l, \chi_{p_i}^l) = \mathbf{GCP}_p^l(n_i^l, \mathcal{F}_{ij}) \quad (8)$$

$$x_i^l = x_i^{l-1} + \chi_{p_i}^l, \text{where } \chi_{p_i}^l \in \mathbb{R}^{1\times 3}. \quad (9)$$

B. Proofs.

Proof of Proposition 1.

Proof. Suppose the vector-valued features given to the corresponding **GCPConv** layers in GCPNet are node features χ_i and edge features ξ_{ij} that are O(3)-equivariant (i.e., 3D rotation and reflection-equivariant) by way of their construction. Additionally, suppose the scalar-valued features given to the respective **GCPConv** layers in GCPNet are E(3)-invariant (i.e., 3D rotation, reflection, and translation-invariant) node features h_i and edge features e_{ij}.

Translation equivariance. In line with Du et al. (2022), the **Centralize** operation on Line 2 of Algorithm 1 in the main text first ensures that $\mathbf{X}^0$ becomes 3D translation invariant by the following procedure. Let $\mathbf{X}(t) = (\mathbf{x}_1(t), ..., \mathbf{x}_n(t))$ represent a many-body system at time t, where the centroid of the system is defined as

$$C(t) = \frac{\mathbf{x}_1(t) + ... + \mathbf{x}_n(t)}{n}. \quad (10)$$

Note that in uniformly translating the position of the system by a vector $\mathbf{v}$, we have $\mathbf{X}(t)+\mathbf{v} \longrightarrow C(t)+\mathbf{v}$, meaning that the centroid of the system translates in the same manner as the system itself. However, note that if at time $t = 0$ we recenter the origin of $\mathbf{X}$ to its centroid, we have

$$\mathbf{X}(t) - C(0) \xrightarrow{\text{translation by } \mathbf{v} \text{ at } t=0} \mathbf{X}(t) - C(0)$$

which implies the system $\mathbf{X}$ is translation-<u>invariant</u> under the centralized reference $\mathbf{X}(t) - C(0)$ when the translation vector

$\mathbf{v}$ is applied to $\mathbf{X}$ at time $t = 0$. Concretely, in the case of translation-invariant tasks such as predicting molecular properties or classifying point clouds, here we have successfully achieved 3D translation invariance. Moreover, for translation-equivariant tasks such as forecasting the positions of a many-body system, we can achieve translation equivariance by simply adding $C(0)$ back to the predicted positions. Therefore, using the above methodology, GCPNets are translation equivariant.

Permutation equivariance. Succinctly, we note that since GCPNet operates on graph-structured input data, permutation equivariance is guaranteed by design. For further discussion of why our proposed method as well as why other graph-based algorithms proposed previously are inherently permutation-equivariant, we refer readers to Zaheer et al. (2017). Therefore, GCPNets are permutation-equivariant.

SO(3)-equivariant frames. On Line 3 of Algorithm 1 in the main text, the **Localize** operation constructs SO(3)-equivariant (i.e., 3D rotation-equivariant) frames $\mathcal{F}_{ij}$ in the following manner.

Define our frame encodings as

$$\mathcal{F}_{ij}^t = (a_{ij}^t, b_{ij}^t, c_{ij}^t), \tag{11}$$

where we have

$$a_{ij}^t = \frac{x_i^t - x_j^t}{\|x_i^t - x_j^t\|}, b_{ij}^t = \frac{x_i^t \times x_j^t}{\|x_i^t \times x_j^t\|}, c_{ij}^t = a_{ij}^t \times b_{ij}^t. \tag{12}$$

The proof that $\mathcal{F}_{ij}^t$ is equivariant under SO(3) transformations of its input space is included in Du et al. (2022). However, for completeness, we include a version of it here.

Let $g \in SO(3)$ be an action under which the positions in X transform equivariantly, and $\mathcal{F}_{ij}^t$ be defined as we have it in Equation 11 above. That is, we have

$$(\mathbf{x}_1(t), ..., \mathbf{x}_n(t)) \xrightarrow{g} (g\mathbf{x}_1(t), ..., g\mathbf{x}_n(t)),$$

where from the definition of a_{ij}^t in Equation 12 we have

$$a_{ij}^t \xrightarrow{g} ga_{ij}^t.$$

Considering b_{ij}^t, from Equation 12 we have

$$\begin{aligned}(gx_i(t)) \times (gx_j(t)) &= \det(g)(g^T)^{-1}(x_i(t) \times x_j(t)) \\ &= g(x_i(t) \times x_j(t)),\end{aligned} \tag{13}$$

where using $g^{-1} = g^T$ for the orthogonal matrix g gives us Equation 13. Consequently, $b_{ij}^t \xrightarrow{g} gb_{ij}^t$. Lastly, by applying Equation 13 once again, we have that $c_{ij}^t \xrightarrow{g} gc_{ij}^t$.

Moreover, note that under reflections of x, we have $R : x \to -x$ which gives us $a_{ij}^t \to -a_{ij}^t$. Thereafter, by the right-hand rule, the cross product of two equivariant vectors gives us a pseudo-vector $b_{ij}^t = x_i^t \times x_j^t \to b_{ij}^t$, where subsequently it is implied that $c_{ij}^t \to -c_{ij}^t$. Consequently, we have $\det(-a_{ij}^t, b_{ij}^t, -c_{ij}^t) = 1$, informing us that the frame encodings $\mathcal{F}_{ij}^t$ are rotation-equivariant yet not reflection-equivariant (a symmetry that is important to not enforce when learning representations of chiral molecules such as proteins). Therefore, the frame encodings within GCPNet are SO(3)-equivariant.

Note, after the construction of these frames, that they are used on Line 4 of Algorithm 1 in the main text to embed all node and edge features (i.e., h_i, e_{ij}, χ_i, and ξ_{ij}) using a single **GCP** module as well as in all subsequent **GCP** modules. We will now prove that the feature updates each **GCP** module makes with the frame encodings $\mathcal{F}_{ij}^t$ defined in Equation 11 are SO(3)-equivariant.

SO(3)-equivariant GCP module. The operations of a **GCP** module are illustrated in Figure 2 in the main text and derived in Section 1.2.1 in the main text. Their SO(3) invariance for scalar feature updates and SO(3) equivariance for vector-valued feature updates is proven as follows.

Following the proof of O(3) equivariance for the GVP module in Jing et al. (2020), the proof of SO(3) equivariance within the **GCP** module is similar, with the following modifications. Within the **GCP** module, the vector-valued features (processed separately for nodes and edges) are fed not only through a bottleneck block comprised of downward and upward projection matrices $\mathbf{D_z}$ and $\mathbf{U_z}$ but are also fed into a dedicated downward projection matrix $\mathbf{D}_{\mathcal{S}}$. The output of matrix multiplication between O(3)-equivariant vector features and $\mathbf{D}_{\mathcal{S}}$ yields O(3)-equivariant vector features $v_{i_{\mathcal{S}}}$ that are used as unique inputs for an SO(3)-invariant scalarization operation. In particular, the following demonstrates the invariance of our design for matrix multiplication with our **GCP** module's projection matrices (e.g., $\mathbf{D}_{\mathcal{S}}$). Suppose $\mathbf{W}_h \in \mathbb{R}^{h \times v}$, $\mathbf{V} \in \mathbb{R}^{v \times 3}$, and $\mathbf{Q} \in SO(3) \in \mathbb{R}^{3 \times 3}$. In line with Jing et al. (2020), observe for $\mathbf{D} = (\mathbf{Q}\mathbf{V}^{\mathrm{T}}) \in \mathbb{R}^{3 \times v}$ that

$$\|\mathbf{W}_h \mathbf{D}^{\mathrm{T}}\|_2 = \|\mathbf{W}_h (\mathbf{V}^{\mathrm{T}})^{\mathrm{T}}\|_2 = \|\mathbf{W}_h \mathbf{V}\|_2.$$

Specifically, our SO(3)-invariant scalarization operation is defined as

$$q_{ij} = (v_{i_{\mathcal{S}}} \cdot a_{ij}^t, v_{i_{\mathcal{S}}} \cdot b_{ij}^t, v_{i_{\mathcal{S}}} \cdot c_{ij}^t), \tag{14}$$

where $\mathcal{F}_{ij}^t = (a_{ij}^t, b_{ij}^t, c_{ij}^t)$ denotes the SO(3)-equivariant frame encodings defined in Equations 11 and 12.

To prove that Equation 14 yields SO(3)-invariant scalar features, let $g \in SO(3)$ be an arbitrary orthogonal transformation. Then we have $v_{i_{\mathcal{S}}} \to gv_{i_{\mathcal{S}}}$, and similarly $\mathcal{F}_{ij}^t = (a_{ij}^t, b_{ij}^t, c_{ij}^t) \to (ga_{ij}^t, gb_{ij}^t, gc_{ij}^t)$. Now, similar to Du et al. (2022), we can derive that Equation 14 becomes

$$\begin{aligned}&(v_{i_{\mathcal{S}}} \cdot a_{ij}^t, v_{i_{\mathcal{S}}} \cdot b_{ij}^t, v_{i_{\mathcal{S}}} \cdot c_{ij}^t) \\ &\quad \to ((v_{i_{\mathcal{S}}})^T g^T g a_{ij}^t, (v_{i_{\mathcal{S}}})^T g^T g b_{ij}^t, (v_{i_{\mathcal{S}}})^T g^T g c_{ij}^t) \\ &\quad = (v_{i_{\mathcal{S}}} \cdot a_{ij}^t, v_{i_{\mathcal{S}}} \cdot b_{ij}^t, v_{i_{\mathcal{S}}} \cdot c_{ij}^t),\end{aligned} \tag{15}$$

where we used the fact that $g^T g = I$ due to the orthogonality of g (with I being the identity matrix). Therefore, the scalarization operation proposed in Equation 14, and previously in Equation 3 in the main text (in an alternative form), yields SO(3)-invariant scalars, which is in line with the results of Qiao et al. (2022).

The output of Equation 14, q_{ij}, is then aggregated in Equation 4 in the main text and concatenated in Equation 5 in the main text with the **GCP** module's remaining O(3)-invariant scalar features (i.e., L_2 vector norm features). Note that introducing SO(3)-invariant scalar information into the **GCP** module in this way breaks the 3D reflection symmetry that previous geometric graph convolution modules enforced (Jing et al., 2020), now giving rise within the **GCP** module to SO(3)-invariant and SO(3)-equivariant updates to scalar and vector-valued features, respectively. Therefore, scalar and vector-valued feature updates for nodes and edges within the **GCP** module are SO(3)-invariant and SO(3)-equivariant, respectively.

As in Section A.2, we now turn to discuss the operations within a single **GCPConv** layer, in particular proving that they maintain

the respective SO(3) invariance and SO(3) equivariance for scalar and vector-valued features that the **GCP** module provides.

SO(3)-equivariant GCPConv layer. Via the corresponding proof in Jing et al. (2020), by way of induction all such operations in Equations 1-6 are respectively SE(3)-invariant and SO(3)-equivariant for features $m_{ij}^l = (m_{e_{ij}}^l, m_{\xi_{ij}}^l)$. Thereby, so are features $n_i^l = (h_i^l, \chi_i^l)$, given that the proof of equivariance for the equivariant **LayerNorm** and **Dropout** operations employed within each **GCPConv** has previously been concretized by Jing et al. (2020). Equation 8 concludes the operations of a single **GCPConv** layer by, as desired, updating the positions of each node i in the 3D input graph. To do so, **GCPConv** residually updates current node positions x_i^{l-1} using SO(3)-equivariant vector-valued features $\chi_{p_i}^l$. Therefore, **GCPConv** layers are SO(3)-invariant for scalar feature updates and SO(3)-equivariant for vector-valued node position and feature updates.

SE(3)-equivariant GCPNet. Lastly, as desired, Line 10 of Algorithm 1 in the main text adds $C(0)$ back to the predicted node positions $\mathbf{X}^l$ as provided by each **GCPConv** layer, ultimately imbuing position updates within $\mathbf{X}^l$ with SE(3) equivariance. Line 14 then concludes GCPNet by using the latest frame encodings $\mathcal{F}_{ij}^t$ to perform, as desired, a final SO(3)-invariant and SO(3)-equivariant projection for scalar and vector-valued features, respectively. Therefore, as desired, GCPNets are SE(3)-invariant for scalar feature updates, SE(3)-equivariant for vector-valued node position and feature updates, and, as a consequence, satisfy the constraint proposed in Def. 1 of the main text.

□

Proof of Proposition 2.

Proof. The proof of SE(3) invariance for scalar node and edge features, h_i and e_{ij}, follows as a corollary of Appendix B.1 (**SE(3)-equivariant GCPNet**). Therefore, GCPNets are SE(3)-invariant concerning their predicted scalar node and edge features and, as a consequence, are geometrically self-consistent according to the constraint in Def. 2 of the main text.

□

Proof of Proposition 3.

Proof. Suppose that GCPNet designates its local geometric representation for layer t to be $\mathcal{F}_{ij}^t = (a_{ij}^t, b_{ij}^t, c_{ij}^t)$, where $a_{ij}^t = \frac{x_i^t - x_j^t}{\|x_i^t - x_j^t\|}, b_{ij}^t = \frac{x_i^t \times x_j^t}{\|x_i^t \times x_j^t\|}$, and $c_{ij}^t = a_{ij}^t \times b_{ij}^t$, respectively. As in Du et al. (2022), this formulation of $\mathcal{F}_{ij}^t$ is proven in Appendix B.1 (*SO(3)-equivariant frames*) to be an SO(3)-equivariant local orthonormal basis at the tangent space of x_i^t and is thereby geometrically complete. Note this implies that GCPNet permits no loss of geometric information as discussed in Appendix A.5 of Du et al. (2022). Therefore, GCPNets are geometry-complete and satisfy the constraint proposed in Def. 3 of the main text.

□

C. Additional Experiments and Results.

In this section, we explore two additional modeling tasks, computational protein design and protein-ligand binding affinity prediction with known or predicted protein-ligand interaction (PLI) inputs, with corresponding implementation details discussed in Appendix D.

CPD, Node Classification. Computational protein design (CPD) investigates a method's ability to design native-like protein sequences. In our CPD experiments, we explore fixed-backbone sequence design, where methods are provided with the 3D backbone structure of a protein and asked to generate a corresponding sequence. We train and evaluate each CPD method on the CATH 4.2 dataset created by Ingraham et al. (2019). This dataset contains 18,204, 608, and 1,120 training, validation, and test proteins, respectively, where all available protein structures with 40% nonredundancy are partitioned by their CATH (class, architecture, topology/fold, homologous superfamily) classification. Baseline comparison methods for this task include a mixture of state-of-the-art Transformers, GNNs, and ENNs.

Under the assumption that native sequences are optimized for their structures (Kuhlman and Baker, 2000), the metrics with which we evaluate each method measure how well a method can distinguish a native-like sequence from a non-native sequence. In particular, following Ingraham et al. (2019), we adopt model perplexity as a measure of how well a method can model the language of native protein sequences. Similarly, we employ native sequence recovery (i.e., amino acid recovery) rates as a way of evaluating, on average, how well each method can design sequences that resemble native protein sequences.

Table 1 shows that, in representing proteins as amino acid residue-level graphs, GCPNet matches or exceeds the performance of several state-of-the-art prediction methods for CPD. In particular, GCPNet improves upon state-of-the-art short sequence recovery rates of previous methods by 0.5% on average while maintaining competitive performance against other methods in all other metrics. We note that all CPD methods marked with * perform model inference autoregressively, introducing a significant computational bottleneck for real-world applications of these models. Inference with GCPNet, in contrast, is designed for direct prediction of amino acid sequences corresponding to a 3D protein structure, thereby decreasing inference runtime by more than a factor of two compared to other methods. While being a simple direct-shot prediction method for CPD, GCPNet is still able to achieve competitive results in terms of amino acid recovery rates for sequence generation, with reasonable results in terms of perplexity as well.

Interestingly, in the context of CPD, an ablation of our equivariant local frames $\mathcal{F}_{ij}$ reveals that such frames are not useful for increasing GCPNet's confidence in its structural understanding of the language of proteins (i.e., its perplexity). This suggests that future work could involve exploring alternative geometric encoding schemes for residue-based graphs when approaching the CPD task (Gao et al., 2022) with GCPNet. This finding highlights the fact that the local frames $\mathcal{F}_{ij}$ appear to be most useful in the context of representation learning on atomic graphs where lower-level molecular motifs are likely to appear, implying that future work towards improving CPD results with GCPNet could involve developing novel atom-level encoding schemes for residue-based graph predictions to leverage the promising results GCPNet yields in other dataset contexts. Nonetheless, our remaining ablations demonstrate that other design characteristics of GCPNet such as the **ResGCP** module and scalar and vector-valued feature representations enable GCPNet to better decode sequence-based information from 3D protein structures.

Table 1. Comparison of GCPNet with baseline methods for the CPD task. Results are reported in terms of the perplexity and amino acid recovery rates of each method for fixed-backbone sequence design. The best results for this task are in **bold**, and the second-best results are underlined.

	Perplexity ↓			Recovery ↑		
Method	Short	Single	All	Short	Single	All
STran* (Ingraham et al., 2019)	8.54	9.03	6.85	28.30	27.60	36.40
SGNN* (Jing et al., 2020)	8.31	8.88	6.55	28.40	28.10	37.30
GVP* (Jing et al., 2021)	7.10	7.44	5.29	32.10	32.00	40.20
GBP* (Aykent and Xia, 2022)	**6.14**	**6.46**	**5.03**	33.22	**33.22**	**42.70**
GCPNet w/o Frames	7.71	8.18	5.87	31.82	31.72	41.18
GCPNet w/o ResGCP	9.63	9.88	7.09	27.27	27.02	35.27
GCPNet w/o Scalars	18.51	18.37	18.17	8.70	8.55	8.62
GCPNet w/o Vectors	10.41	10.53	8.87	26.42	26.23	28.99
GCPNet	8.22	8.60	6.06	**33.33**	32.86	40.32

Table 2. Comparison of GCPNet with baseline methods for the LBA task, with new baselines featuring known or predicted-PLI inputs. The results are averaged over three independent runs, with each method being cross-validated on the ATOM3D benchmark's PDBBind "refined set" (v.2019) (Townshend et al., 2020) unless otherwise specified. The top-1 (best) results for this task are in **bold**, and the second-best results are underlined. - denotes a metric value that is unavailable, and † denotes a method that is instead cross-validated on the PDBBind "core set" (v.2016).

Type	Method	RMSE ↓	p ↑	Sp ↑
CNN	3DCNN (Wang et al., 2023b)	1.416 ± 0.021	0.550	0.553
	DeepDTA (Wang et al., 2023b)	1.866 ± 0.080	0.472	0.471
	DeepAffinity (Aykent and Xia, 2022)	1.893 ± 0.650	0.415	0.426
RNN	Bepler and Berger (Wang et al., 2023b)	1.985 ± 0.006	0.165	0.152
	TAPE (Wang et al., 2023b)	1.890 ± 0.035	0.338	0.286
	ProtTrans (Wang et al., 2023b)	1.544 ± 0.015	0.438	0.434
GNN	GCN (Wang et al., 2023b)	1.601 ± 0.048	0.545	0.533
	DGAT (Aykent and Xia, 2022)	1.719 ± 0.047	0.464	0.472
	DGIN (Aykent and Xia, 2022)	1.765 ± 0.076	0.426	0.432
	DGAT-GCN (Aykent and Xia, 2022)	1.550 ± 0.017	0.498	0.496
	MaSIF (Wang et al., 2023b)	1.484 ± 0.018	0.467	0.455
	IEConv (Wang et al., 2023b)	1.554 ± 0.016	0.414	0.428
	Holoprot-Full Surface (Wang et al., 2023b)	1.464 ± 0.006	0.509	0.500
	Holoprot-Superpixel (Wang et al., 2023b)	1.491 ± 0.004	0.491	0.482
	ProNet-Amino-Acid (Wang et al., 2023b)	1.455 ± 0.009	0.536	0.526
	ProNet-Backbone (Wang et al., 2023b)	1.458 ± 0.003	0.546	0.550
	ProNet-All-Atom (Wang et al., 2023b)	1.463 ± 0.001	0.551	0.551
	GeoSSL-DDM (Liu et al., 2023)	1.451 ± 0.030	0.577	0.572
ENN	Cormorant (Aykent and Xia, 2022)	1.568 ± 0.012	0.389	0.408
	PaiNN	1.698 ± 0.050	0.366	0.358
	ET	1.490 ± 0.019	0.564	0.532
	GVP (Aykent and Xia, 2022)	1.594 ± 0.073	0.434	0.432
	GBP (Aykent and Xia, 2022)	1.405 ± 0.009	0.561	0.557
Known-PLI	OnionNet† (Zheng et al., 2019)	1.278	0.816	-
	FAST† (Jones et al., 2021)	1.308	0.810	-
	DeepDTAF† (Wang et al., 2021b)	1.355	0.789	-
	CAPLA† (Jin et al., 2023)	**1.200**	**0.843**	-
Predicted-PLI	CAPLA-Pred† (Jin et al., 2023)	1.298	0.825	-
Ours	GCPNet w/o Frames	1.485 ± 0.015	0.521	0.504
	GCPNet w/o ResGCP	1.514 ± 0.008	0.471	0.468
	GCPNet w/o Scalars	1.685 ± 0.000	0.050	0.000
	GCPNet w/o Vectors	1.727 ± 0.005	0.270	0.304
	GCPNet	1.352 ± 0.003	0.608	**0.607**

LBA, Graph Classification with Known-PLI Inputs. To investigate the impact of incorporating protein-ligand interactions (e.g., binding pockets) into one's prediction method for protein-ligand binding affinity, here we expand our selection of baseline methods for the LBA task. Table 2 shows that state-of-the-art methods that utilize either known or predicted-PLI information to predict the binding affinity of a protein-ligand complex surpass the performance of GCPNet for the LBA task in terms of RMSE and Pearson's correlation. Note that (1) Table 2 in the main text does not feature these additional baseline methods to enable direct and fair comparisons between the methods listed therein, as all original baseline methods for the LBA task do not make

Table 3. Summary of GCPNet's node and edge features for 3D input graphs derived for the LBA and PSR tasks. Here, N and E denote the number of nodes and edges in $\mathcal{G}$, respectively.

	Feature	Type	Shape
Node Features (h)	One-hot encoding of atom type	Categorical (Scalar)	$N \times 9$
Node Features (χ)	Directional encoding of orientation	Numeric (Vector)	$N \times 2$
Edge Features (e)	Radial basis distance embedding	Numeric (Scalar)	$E \times 16$
Edge Features (ξ)	Pairwise atom position displacement	Numeric (Vector)	$E \times 1$
Total	Node features		$N \times 11$
	Edge features		$E \times 17$

Table 4. Summary of GCPNet's node and edge features for 3D input graphs derived for the CPD task.

	Feature	Type	Shape
Node Features (h)	Dihedral angle encoding	Numeric (Scalar)	$N \times 6$
Node Features (χ)	Orientation and sidechain encoding	Numeric (Vector)	$N \times 3$
Edge Features (e)	Distance and positional embedding	Numeric (Scalar)	$E \times 32$
Edge Features (ξ)	Pairwise atom position displacement	Numeric (Vector)	$E \times 1$
Total	Node features		$N \times 9$
	Edge features		$E \times 33$

use of explicit PLI information; and (2) these additional baseline methods are cross-validated on the PDBBind "core set" (v.2016), whereas the remainder of the baseline methods listed in Table 2 (following the standardized ATOM3D benchmark) (Townshend et al., 2020) are cross-validated on a redundancy-reduced version of the PDBBind "refined set" (v.2019) which permits no more than 30% sequence identity between any protein in the test dataset and any protein in the training or validation datasets. Nonetheless, the results in Table 2 suggest an interesting avenue for future research with GCPNet, in that combining its learned representations with known or predicted-PLI information may yield improved results for the LBA task, following Wang et al. (2023a); Zeng et al. (2023). We defer an exploration of this idea to future work.

D. Implementation Details.

Featurization. As shown in Table 3, for the LBA and PSR tasks, in each 3D input graph, we include as a scalar node feature an atom's type using a 9-dimensional one-hot encoding vector for each atom. As vector-valued node features, we include *forward* and *reverse* unit vectors in the direction of $x_{i+1} - x_i$ and $x_{i-1} - x_i$, respectively (i.e., the node's 3D orientation). For the input 3D graphs' scalar edge features, we encode the distance $\|x_i - x_j\|_2$ using Gaussian radial basis functions, where we use 16 radial basis functions with centers evenly distributed between 0 and 20 units (e.g., Angstrom). For the graphs' vector-valued edge features, we encode the unit vector in the direction of $x_i - x_j$ (i.e., pairwise atom position displacements).

As displayed in Table 4, for the CPD task, in each 3D input graph, we include as scalar node features an encoding of each amino acid residue's dihedral angles $\{\sin, \cos\} \circ \{\phi, \psi, \omega\}$, where $\mathbf{\Phi}$, ψ, and ω are the dihedral angles computed from the corresponding protein's C_{i-1}, N_i, C_i, and N_{i+1} backbone atoms. We then include as vector-valued node features each node's 3D orientation. For edge features, we use Gaussian radial basis function distance encodings as scalar edge features and pairwise atom position displacements as vector-valued edge features.

As illustrated in Table 5, for the NMS task, in each 3D input graph, we include as a scalar node feature an invariant encoding of each node's velocity vector, namely $\sqrt{v_i^2}$. Each node's velocity and orientation are encoded as vector-valued node features. Scalar edge features are represented as Gaussian radial basis distance encodings as well as the product of the charges in each node pair (i.e., $c_i c_j$). Lastly, vector-valued edge features are represented as pairwise atom position displacements.

Hardware Used. The Oak Ridge Leadership Facility (OLCF) at the Oak Ridge National Laboratory (ORNL) is an open science computing facility that supports HPC research. The OLCF houses the Summit compute cluster. Summit, launched in 2018, delivers 8 times the computational performance of Titan's 18,688 nodes, using only 4,608 nodes. Like Titan, Summit has a hybrid architecture, and each node contains multiple IBM POWER9 CPUs and NVIDIA Volta GPUs all connected with NVIDIA's high-speed NVLink. Each node has over half a terabyte of coherent memory (high bandwidth memory + DDR4) addressable by all CPUs and GPUs plus 800GB of non-volatile RAM that can be used as a burst buffer or as extended memory. To provide a high rate of I/O throughput, the nodes are connected in a non-blocking fat-tree using a dual-rail Mellanox EDR InfiniBand interconnect. We used the Summit compute cluster to train all our models. For the LBA and NMS tasks, we used 16GB NVIDIA Tesla V100 GPUs for model training, whereas for the memory-intensive PSR and CPD tasks, we used 32GB V100 GPUs instead.

Software Used. We used Python 3.8.12 (Van Rossum and Drake, 2009), PyTorch 1.10.2 (Paszke et al., 2019), PyTorch Lightning 1.7.7 (Falcon, 2019), and PyTorch Geometric 2.1.0post0 (Fey and Lenssen, 2019) to run our deep learning experiments. For each model trained, PyTorch Lightning was used to facilitate model checkpointing, metrics reporting, and distributed data parallelism across 6 V100 GPUs. A more in-depth description of the software environment used to train and run inference with our models is available at https://github.com/BioinfoMachineLearning/GCPNet.

Table 5. Summary of GCPNet's node and edge features for 3D input graphs derived for the NMS task.

	Feature	Type	Shape
Node Features (h)	Invariant velocity encoding	Numeric (Scalar)	$N \times 1$
Node Features (χ)	Velocity and orientation encoding	Numeric (Vector)	$N \times 3$
Edge Features (e)	Edge and distance embedding	Numeric (Scalar)	$E \times 17$
Edge Features (ξ)	Pairwise atom position displacement	Numeric (Vector)	$E \times 1$
Total	Node features		$N \times 4$
	Edge features		$E \times 18$

Hyperparameters. As shown in Tables 6, 7, 8, 9, and 10, we use a learning rate of 10^{-4} with GCPNet for all tasks besides the RS task. The learning rate is kept constant throughout each model's training. For the NMS task, each model is trained for a minimum of 100 epochs and a maximum of 12,000 epochs. For all other tasks, each model is trained for a minimum of 100 epochs and a maximum of 1,000 epochs. For a given task, models with the best loss on the corresponding validation data split are then tested on the test split for the respective task. Note that, for the RS task, we do not perform any model hyperparameter tuning, following previous conventions from Schneuing et al. (2023). Test set run times are listed in Table 11 for each task using the corresponding hyperparameter-tuned GCPNet model.

E. Representation Learning of 3D Biomolecules.

Comparison to Existing Protein Representation Learning Methods.

In Table 12, we compare GCPNet to previous protein representation learning methods to highlight its distinguishing capabilities. In particular, GCPNet is the only method that can produce arbitrary vector outputs while respecting SE(3) symmetries and, consequently, full sensitivity to molecular chirality. Furthermore, it can do so while representing 3D protein structures completely and self-consistently, thereby with no loss of force-related or geometric information.

Future Directions for Representation Learning of 3D Biomolecules.

Towards enhanced geometric representation learning of 3D biomolecules, we postulate that chirality sensitivity may be further strengthened for downstream tasks via a chirality-specific auxiliary loss function employed during model training. For example, drawing inspiration from AlphaFold 2 (Jumper et al., 2021), such a loss function may periodically (e.g., 50% of the time) penalize a method for producing scalar graph representations of mirrored biomolecular structures that are highly similar to one another in terms of cosine vector similarity, although doing so would require two forward passes of the model (e.g., 50% of the time) during training. Future work may investigate the utility of such auxiliary training objectives or efficient proxies of them.

To strengthen a method's awareness of global forces, we believe future research into optimal strategies for dynamically updating local coordinate frames during a method's forward pass may prove useful for geometric representation learning of atomic systems. For example, updating a method's local coordinate frames between individual network layers that directly update node coordinates may yield fruitful results in this direction. Consequently, such techniques warrant further investigation in future work.

Lastly, in light of the promising results with GCPNet in the Results section of the main text, future work on the model could involve researching more computationally efficient variations of GCPNet that require fewer GCP message-passing layers within each GCP convolution layer or that embed geometric frames sparsely rather than in each GCP layer. For the LBA task in particular, incorporating known protein-ligand interaction information to predict binding affinity is a promising direction for future work on binding affinity prediction (Wang et al., 2021a) and may lead to improved performance for many of the methods listed in Table 2 of the main text. Another promising future direction to improve methods such as GCPNet is to improve the expressivity of such methods by learning higher-order equivariant tensors within one's message-passing procedure. Enhancing geometric

Table 6. Hyperparameters used with all GCPNet models for the RS task.

Hyperparameter	Selected Values
Number of GCPNet Layers	**8**
Number of **GCP** Message-Passing Layers	**8**
χ Hidden Dimensionality	**16**
Learning Rate	**0.0005**
Weight Decay Rate	**0**
**GCP** Dropout Rate	**0.1**
Dense Layer Dropout Rate	**0.1**

Table 7. Hyperparameter search space for all GCPNet models through which we searched to obtain strong performance on the LBA task's validation split. The final parameters for the standard GCPNet model for the LBA task are in **bold**.

Hyperparameter	Search Space
Number of GCPNet Layers	7, **8**
Number of **GCP** Message-Passing Layers	**8**
χ Hidden Dimensionality	**16**, 32
Learning Rate	**0.0001**, 0.0003
Weight Decay Rate	**0**
**GCP** Dropout Rate	**0.1**, 0.25
Dense Layer Dropout Rate	**0.1**, 0.25

expressiveness to thereby increase a method's effective run time efficiency would allow GCPNet to be used increasingly in new scientific and deep learning applications requiring high computational throughput (e.g., virtual screening of new drugs).

References

Aykent, S. and Xia, T. Gbpnet: Universal geometric representation learning on protein structures. In *Proceedings of the 28th ACM SIGKDD Conference on Knowledge Discovery and Data Mining*, KDD '22, page 4–14, New York, NY, USA, 2022. Association for Computing Machinery. ISBN 9781450393850. doi: 10.1145/3534678.3539441. URL `https://doi.org/10.1145/3534678.3539441`.

Brandstetter, J. et al. Geometric and physical quantities improve e (3) equivariant message passing. *arXiv preprint arXiv:2110.02905*, 2021.

Du, W. et al. SE(3) equivariant graph neural networks with complete local frames. In Chaudhuri, K. et al, editors, *Proceedings of the 39th International Conference on Machine Learning*, volume 162 of *Proceedings of Machine Learning Research*, pages 5583–5608. PMLR, 2022. URL `https://proceedings.mlr.press/v162/du22e.html`.

Falcon, WA, e.a. Pytorch lightning. *https://github.com/PyTorchLightning/pytorch-lightning*, 3, 2019.

Fey, M. and Lenssen, J.E. Fast graph representation learning with pytorch geometric. *ArXiv*, abs/1903.02428, 2019.

Gao, Z. et al. Pifold: Toward effective and efficient protein inverse folding. *arXiv preprint arXiv:2209.12643*, 2022.

Hermosilla, P. et al. Intrinsic-extrinsic convolution and pooling for learning on 3d protein structures. In *International Conference on Learning Representations*, 2021. URL `https://openreview.net/forum?id=l0mSUROpwY`.

Ingraham, J. et al. Generative models for graph-based protein design. *Advances in neural information processing systems*, 32, 2019.

Jin, Z. et al. Capla: improved prediction of protein–ligand binding affinity by a deep learning approach based on a cross-attention mechanism. *Bioinformatics*, 39(2):btad049, 2023.

Jing, B. et al. Learning from protein structure with geometric vector perceptrons. *arXiv preprint arXiv:2009.01411*, 2020.

Jing, B. et al. Equivariant graph neural networks for 3d macromolecular structure. *arXiv preprint arXiv:2106.03843*, 2021.

Jones, D. et al. Improved protein–ligand binding affinity prediction with structure-based deep fusion inference. *Journal of chemical information and modeling*, 61(4):1583–1592, 2021.

Jumper, J. et al. Highly accurate protein structure prediction with alphafold. *Nature*, 596(7873):583–589, 2021.

Kuhlman, B. and Baker, D. Native protein sequences are close to optimal for their structures. *Proceedings of the National Academy of Sciences*, 97(19):10383–10388, 2000.

Liu, S. et al. Molecular geometry pretraining with SE(3)-invariant denoising distance matching. In *The Eleventh International Conference on Learning Representations*, 2023. URL `https://openreview.net/forum?id=CjTHVo1dvR`.

Morehead, A. et al. Geometric transformers for protein interface contact prediction. In *International Conference on Learning Representations*, 2022. URL `https://openreview.net/forum?id=CS4463zx6Hi`.

Paszke, A. et al. Pytorch: An imperative style, high-performance deep learning library. In Wallach, H. et al, editors, *Advances in Neural Information Processing Systems 32*, pages 8024–8035. Curran Associates, Inc., 2019.

Qiao, Z. et al. Dynamic-backbone protein-ligand structure prediction with multiscale generative diffusion models. *arXiv preprint arXiv:2209.15171*, 2022.

Schneuing, A. et al. Structure-based drug design with equivariant diffusion models, 2023. URL `https://openreview.net/forum?id=uKmuzIuVl8z`.

Townshend, R.J. et al. Atom3d: Tasks on molecules in three dimensions. *arXiv preprint arXiv:2012.04035*, 2020.

Table 8. Hyperparameter search space for all GCPNet models through which we searched to obtain strong performance on the PSR task's validation split. The final parameters for the standard GCPNet model for the PSR task are in **bold**.

Hyperparameter	Search Space
Number of GCPNet Layers	**5**
Number of **GCP** Message-Passing Layers	**8**
χ Hidden Dimensionality	**16**, 32
Learning Rate	**0.0001**, 0.0003
Weight Decay Rate	**0**, 0.0001
**GCP** Dropout Rate	**0.1**, 0.25
Dense Layer Dropout Rate	**0.1**, 0.25

Table 9. Hyperparameter search space for all GCPNet models through which we searched to obtain strong performance on the CPD task's validation split. The final parameters for the standard GCPNet model for the CPD task are in **bold**.

Hyperparameter	Search Space
Number of GCPNet Encoder Layers	**9**
Number of GCPNet Decoder Layers	**3**
Number of **GCP** Message-Passing Layers	**8**
χ Hidden Dimensionality	**16**, 32
Learning Rate	**0.0001**
Weight Decay Rate	0.0, $\mathbf{10^{-8}}$, 0.0001
**GCP** Dropout Rate	0.1, **0.2**, 0.25, 0.4
Decoder Residual Updates	False, **True**

Van Rossum, G. and Drake, F.L. *Python 3 Reference Manual*. CreateSpace, Scotts Valley, CA, 2009. ISBN 1441412697.

Wang, D.D. et al. Structure-based protein–ligand interaction fingerprints for binding affinity prediction. *Computational and Structural Biotechnology Journal*, 19:6291–6300, 2021a.

Wang, K. et al. Deepdtaf: a deep learning method to predict protein–ligand binding affinity. *Briefings in Bioinformatics*, 22 (5):bbab072, 2021b.

Wang, K. et al. Graphscoredta: optimized graph neural network for protein–ligand binding affinity prediction. *Bioinformatics*, 39(6):btad340, 2023a.

Wang, L. et al. Learning hierarchical protein representations via complete 3d graph networks. In *The Eleventh International Conference on Learning Representations*, 2023b. URL `https://openreview.net/forum?id=9X-hgLDLYkQ`.

Zaheer, M. et al. Deep sets. *Advances in neural information processing systems*, 30, 2017.

Zeng, X. et al. Fusing sequence and structural knowledge by heterogeneous models to accurately and interpretively predict drug–target affinity. *Molecules*, 28(24):8005, 2023.

Zhang, Z. et al. Protein representation learning by geometric structure pretraining. *arXiv preprint arXiv:2203.06125*, 2022.

Zheng, L. et al. Onionnet: a multiple-layer intermolecular-contact-based convolutional neural network for protein–ligand binding affinity prediction. *ACS omega*, 4(14):15956–15965, 2019.

Table 10. Hyperparameter search space for all GCPNet models through which we searched to obtain strong performance on the NMS task's validation split. The final parameters for the standard GCPNet model for the NMS task are in **bold**.

Hyperparameter	Search Space
Number of GCPNet Layers	**4**, 7
Number of **GCP** Message-Passing Layers	**8**
χ Hidden Dimensionality	**16**
Learning Rate	**0.0001**, 0.0003
Weight Decay Rate	**0**
**GCP** Dropout Rate	0.0, **0.1**

Table 11. Run times (in seconds) using GCPNet on the test dataset of each task.

Task	Test Dataset Size	Run Time (s)
RS	4480	472
LBA	490	47
PSR	16014	2231
NMS - ES(5)	2000	18
NMS - ES(20)	2000	12
NMS - G+ES(20)	2000	12
NMS - L+ES(20)	2000	12

Table 12. Comparisons of existing protein geometric representation learning methods, adapted from Wang et al. (2023b). Firstly, modeling protein graph nodes as atoms expands the range of molecular functions a method can directly represent (e.g., force field parameters) at the cost of increased computational complexity. Here n, N, and k denote the number of amino acids, the number of atoms, and the average degree in a 3D protein graph, and $N >> n$. Our method is the only one that can learn and produce general vector outputs while maintaining SE(3) symmetries and thereby sensitivity to molecular chirality. Lastly, our method can do so while capturing 3D structures in a geometry-complete (i.e., local coordinates-wise) and self-consistent (i.e., scalar-wise SE(3)-invariant) manner.

Method	Node Type	Complexity	Symmetry	Complete	Self-Consistent	Produces General Vectors	Chirality-Aware
GearNet (Zhang et al., 2022)	Amino Acid	O(nk)	E(3) invariance	✗	✗	✗	✗
ProNet (Wang et al., 2023b)	Amino Acid	O(nk)	SE(3) invariance	✓	✓	✗	✓
GVP-GNN etc. (Jing et al., 2020)	Amino Acid	O(nk)	E(3) equivariance	✗	✗	✓	✗
Vector-Gated GVP-GNN (Jing et al., 2021)	Atom	O(Nk)	E(3) equivariance	✗	✗	✓	✗
IEConv (Hermosilla et al., 2021)	Atom	O(Nk)	E(3) invariance	✗	✗	✗	✗
ClofNet (Du et al., 2022)	Atom	O(Nk)	SE(3) equivariance	✓	✓	✗	✓
Ours	Atom	O(Nk)	SE(3) equivariance	✓	✓	✓	✓
